# Supplementary figures and images for: Using big data to improve cardiovascular care and outcomes in China: a protocol for the CHinese Electronic health Records Research in Yinzhou (CHERRY) Study
Source: BMJ Open. 2018 Feb 12;8(2):e019698. doi: 10.1136/bmjopen-2017-019698 (PMC5829949; doi:10.1136/bmjopen-2017-019698)

Administrative databases for CHERRY cohort

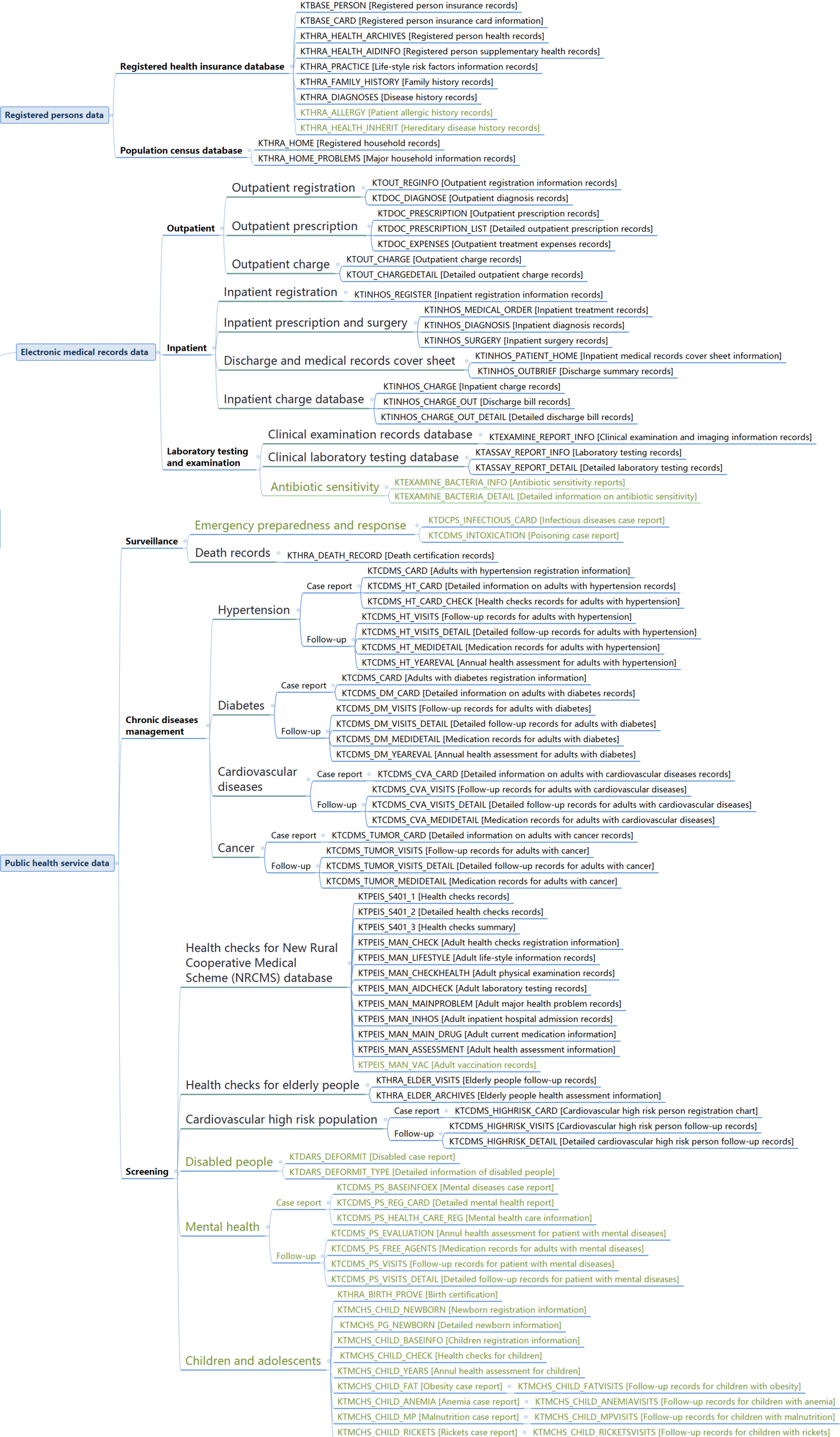

Supplement: Supplementary Figure 1 [file bmjopen-2017-019698supp001.pdf]
