# Supplementary material for: Using big data to improve cardiovascular care and outcomes in China: a protocol for the CHinese Electronic health Records Research in Yinzhou (CHERRY) Study
Source: BMJ Open. 2018 Feb 12;8(2):e019698. doi: 10.1136/bmjopen-2017-019698 (PMC5829949; doi:10.1136/bmjopen-2017-019698)
Supplement: Supplementary Table 1 [file bmjopen-2017-019698supp002.pdf]

Supplemental files

Table S1. Description of CHERRY study data sources and dataset codes

Research databases	Data sources	Dataset codes	Description
Socio-demographics	Registered health insurance database	KTBASE_PERSON	Registered person insurance records
		KTBASE_CARD	Registered person insurance card information
		KTHRA_HEALTH_ARCHIVES	Registered person health records
		KTHRA_HEALTH_AIDINFO	Registered person supplementary health records
		KTHRA_PRACTICE	Life-style risk factors information records
		KTHRA_FAMILY_HISTORY	Family history records
		KTHRA_DIAGNOSES	Disease history records
	Population census database	KTHRA_HOME	Registered household records
		KTHRA_HOME_PROBLEMS	Major household information records

Longitudinal measurements on cardiovascular risk factors	Health checks for New Rural Cooperative Medical Scheme (NRCMS) database	KTPEIS_S401_1	Health checks records
		KTPEIS_S401_2	Detailed health checks records
		KTPEIS_S401_3	Health checks summary
		KTPEIS_MAN_CHECK	Adult health checks registration information
		KTPEIS_MAN_LIFESTYLE	Adult life-style information records
		KTPEIS_MAN_CHECKHEALTH	Adult physical examination records
		KTPEIS_MAN_AIDCHECK	Adult laboratory testing records
		KTPEIS_MAN_MAINPROBLEM	Adult major health problem records
		KTPEIS_MAN_INHOS	Adult inpatient hospital admission records
		KTPEIS_MAN_MAIN_DRUG	Adult current medication information
		KTPEIS_MAN_ASSESSMENT	Adult health assessment information

	Health checks for elderly people database	KTHRA_ELDER_VISITS	Elderly people follow-up records
		KTHRA_ELDER_ARCHIVES	Elderly people health assessment information
	Health checks for adults with hypertension database	KTCDMS_CARD	Adults with hypertension registration information
		KTCDMS_HT_CARD	Detailed information on adults with hypertension records
		KTCDMS_HT_CARD_CHECK	Health checks records for adults with hypertension
		KTCDMS_HT_VISITS	Follow-up records for adults with hypertension
		KTCDMS_HT_VISITS_DETAIL	Detailed follow-up records for adults with hypertension
		KTCDMS_HT_MEDIDETAIL	Medication records for adults with hypertension
		KTCDMS_HT_YEAREVAL	Annual health assessment for adults with hypertension
	Health checks for adults with diabetes database	KTCDMS_CARD	Adults with diabetes registration information
		KTCDMS_DM_CARD	Detailed information on adults with diabetes records

		KTCDMS_DM_VISITS	Follow-up records for adults with diabetes
		KTCDMS_DM_VISITS_DETAIL	Detailed follow-up records for adults with diabetes
		KTCDMS_DM_MEDIDETAIL	Medication records for adults with diabetes
		KTCDMS_DM_YEAREVAL	Annual health assessment for adults with diabetes
	Cardiovascular high-risk population	KTCDMS_HIGHRISK_CARD	Cardiovascular high-risk person registration chart
		KTCDMS_HIGHRISK_VISITS	Cardiovascular high-risk person follow-up records
		KTCDMS_HIGHRISK_DETAIL	Detailed cardiovascular high-risk person follow-up records
	Clinical examination records database	KTEXAMINE_REPORT_INFO	Clinical examination and imaging information records
	Clinical laboratory testing database	KTASSAY_REPORT_INFO	Laboratory testing records
		KTASSAY_REPORT_DETAIL	Detailed laboratory testing records

Health care services and medications	Outpatient medical records database	KTOUT_REGINFO	Outpatient registration information records
		KTDOC_DIAGNOSE	Outpatient diagnosis records
	Outpatient prescription database	KTDOC_PRESCRIPTION	Outpatient prescription records
		KTDOC_PRESCRIPTION_LIST	Detailed outpatient prescription records
		KTDOC_EXPENSES	Outpatient treatment expenses records
	Outpatient charge database	KTOUT_CHARGE	Outpatient charge records
		KTOUT_CHARGEDETAIL	Detailed outpatient charge records
	Inpatient medical records database	KTINHOS_REGISTER	Inpatient registration information records
		KTINHOS_PATIENT_HOME	Inpatient medical records cover sheet information
		KTINHOS_OUTBRIEF	Discharge summary records

	Inpatient prescription database	KTINHOS_MEDICAL_ORDER	Inpatient treatment records
		KTINHOS_DIAGNOSIS	Inpatient diagnosis records
		KTINHOS_SURGERY	Inpatient surgery records
	Inpatient charge database	KTINHOS_CHARGE	Inpatient charge records
		KTINHOS_CHARGE_OUT	Discharge bill records
		KTINHOS_CHARGE_OUT_DETAIL	Detailed discharge bill records
Clinical outcome events	Cardiovascular diseases surveillance and management system	KTCDMS_CVA_CARD	Detailed information on adults with cardiovascular disease records
		KTCDMS_CVA_VISITS	Follow-up records for adults with cardiovascular diseases
		KTCDMS_CVA_VISITS_DETAIL	Detailed follow-up records for adults with cardiovascular diseases
		KTCDMS_CVA_MEDIDETAIL	Medication records for adults with cardiovascular diseases

	Cancer surveillance database	KTCDMS_TUMOR_CARD	Detailed information on adults with cancer records
		KTCDMS_TUMOR_VISITS	Follow-up records for adults with cancer
		KTCDMS_TUMOR_VISITS_DETAIL	Detailed follow-up records for adults with cancer
		KTCDMS_TUMOR_MEDIDETAIL	Medication records for adults with cancer
	Database of death records	KTHRA_DEATH_RECORD	Death certificates
