# Supplementary material for: Using big data to improve cardiovascular care and outcomes in China: a protocol for the CHinese Electronic health Records Research in Yinzhou (CHERRY) Study
Source: BMJ Open. 2018 Feb 12;8(2):e019698. doi: 10.1136/bmjopen-2017-019698 (PMC5829949; doi:10.1136/bmjopen-2017-019698)
Supplement: Supplementary Table 2 [file bmjopen-2017-019698supp003.pdf]

Table S2. Information on cardiovascular disease diagnosis in different data sources for the CHERRY study

	Clinical diagnosis	ICD-10 Code	Clinical symptom		Neurological examination	Cerebrospinal fluid (CSF) analysis	Serum enzyme tests	Electrocardiogram (ECG)	Electroencephalogram (EEG)	Coronary computed tomography angiography (CTA)	Computed tomography (CT) / magnetic resonance imaging (MRI)		Autopsy
			Brief	Detail							Summary	Imaging	
Disease surveillance / Death certificates (gold standard)	√	√	√				√	√	√	√	√		√
Disease management database (primary care)	√	√	√	√			√	√	√		√		
Electronic medical records database (hospital care)	√	√	√	√	√	√	√	√	√	√	√	√	
Health insurance database	√	√											
