# Supplementary material for: Using big data to improve cardiovascular care and outcomes in China: a protocol for the CHinese Electronic health Records Research in Yinzhou (CHERRY) Study
Source: BMJ Open. 2018 Feb 12;8(2):e019698. doi: 10.1136/bmjopen-2017-019698 (PMC5829949; doi:10.1136/bmjopen-2017-019698)
Supplement: Author's manuscript [file bmjopen-2017-019698.draft_revisions.pdf]

BMJ Open is committed to open peer review. As part of this commitment we make the peer review history of every article we publish publicly available.

When an article is published we post the peer reviewers' comments and the authors' responses online. We also post the versions of the paper that were used during peer review. These are the versions that the peer review comments apply to.

The versions of the paper that follow are the versions that were submitted during the peer review process. They are not the versions of record or the final published versions. They should not be cited or distributed as the published version of this manuscript.

BMJ Open is an open access journal and the full, final, typeset and author-corrected version of record of the manuscript is available on our site with no access controls, subscription charges or pay-per-view fees (http://bmjopen.bmj.com).

If you have any questions on BMJ Open's open peer review process please email editorial.bmjopen@bmj.com

BMJ Open

Using big data to improve cardiovascular care and outcomes in China: a protocol for the CHinese Electronic health Records Research in Yinzhou (CHERRY) study

Journal:	*BMJ Open*
Manuscript ID	bmjopen-2017-019698
Article Type:	Protocol
Date Submitted by the Author:	19-Sep-2017
Complete List of Authors:	Lin, Hongbo; Yinzhou District Center for Disease Control and Prevention<br>TANG, Xun; Peking University Health Science Center, Department of Epidemiology and Biostatistics<br>Shen, Peng; Yinzhou District Center for Disease Control and Prevention<br>Zhang, Dudan; Peking University Health Science Center, Department of Epidemiology and Biostatistics<br>Wu, Jinguo; Wonders Information Co., Ltd.<br>Zhang, Jingyi; Wonders Information Co., Ltd.<br>Lu, Ping; Wonders Information Co., Ltd.<br>Si, Yaqin; Peking University Health Science Center, Department of Epidemiology and Biostatistics<br>GAO, Pei; Peking University Health Science Center, Department of Epidemiology and Biostatistics
Keywords:	cardiovascular diseases, electronic health records, Chinese

SCHOLARONE™ Manuscripts

Using big data to improve cardiovascular care and outcomes in China: a protocol for the CHinese Electronic health Records Research in Yinzhou (CHERRY) study

Hongbo Lin, [1]* Xun Tang, [2]* Peng Shen, [1] Dudan Zhang, [2] Jinguo Wu, [3] Jingyi Zhang, [3] Ping Lu, [3] Yaqin Si, [2] Pei Gao, [2#]

[1] Yinzhou District Center for Disease Control and Prevention, Ningbo 315100, China,

[2] Department of Epidemiology and Biostatistics, Peking University Health Science Center, Beijing 100191, China,

[3] Wonders Information Co., Ltd., Shanghai 201112, China.

* Drs. Hongbo Lin and Xun Tang contributed equally to this paper.

[#] **Correspondence to:**

Prof. Pei Gao, PhD

Department of Epidemiology and Biostatistics,

Peking University Health Science Center,

38 Xueyuan Road, Beijing 100191, China

Tel/Fax: +86-10-82805642

Email: peigao@bjmu.edu.cn

Total word count: 4908 words, Abstract: 290 words, 2 Figures and 2 Tables, 27 References, 2 Supplemental Materials

For peer review only - http://bmjopen.bmj.com/site/about/guidelines.xhtml

Abstract

Introduction: Electronic Health Records (EHRs) based data is enriched with individual-level longitudinal measurements of information, becoming an increasingly common data source for clinical risk prediction worldwide. However, few EHR-based cohort study in China is available. Harnessing EHRs for research requires a full understanding of data linkages, management, and data quality in large data sets, presenting both unique analytic opportunities and challenges. The purpose of this study is to provide a framework to make a uniquely integrated EHR database in China available for scientific research.

Methods and Analysis: The CHinese Electronic health Records Research in Yinzhou (CHERRY) study will extract individual participant data within the regional health information system in an eastern coastal area of China to establish a longitudinal population-based ambispective cohort study for cardiovascular care and outcomes research. Totally 965,349 Chinese adults aged over 18 years were registered in the health information system in 2009, and 22,501 deaths were observed from 1st January 2009 until 31st December 2014. The study will include information from multiple epidemiological surveys, EHRs for chronic disease management, health administrative, clinical, laboratory, drug, and electronic medical records databases. Follow-up on fatal and non-fatal clinical events is achieved through records linkage to the regional system of disease surveillance points and chronic disease management, based on the diagnostic codes from the international classification of diseases (the tenth revision). The CHERRY study will provide a unique platform and serves as a valuable big data resource to investigate cardiovascular risk prediction and population management for primary and secondary preventions of cardiovascular events in China.

Ethics and dissemination: The CHERRY study has been approved by the Peking

For peer review only

For peer review only - http://bmjopen.bmj.com/site/about/guidelines.xhtml

University Institutional Review Board (IRB00001052-16011) on April 2016. Results of the study will be disseminated through published journal articles, conferences and seminar presentations.

For peer review only

Strengths and limitations of this study

- The Chinese Electronic health Records Research in Yinzhou (CHERRY) study is a large natural population-based observational cohort study by linking big data on integrated individual-level Electronic Health Records (EHRs).
- The CHERRY study is among the first in China to establish the EHR-based research platform for investigating a wide range of important issues on cardiovascular disease primary and secondary preventions in a real-world circumstance.
- The CHERRY study is unique in being able to trace a complete life journey from birth certificates, health checks, primary care, hospitals, disease surveillance and, ultimately, death certificates in nearly 1 million adults in a general Chinese population.
- Missing data and conflicting data might be the main limitations of any EHRs-converted big data research in terms of data quality; however, developments in imputation for missing data within longitudinal cohorts and setup of the priority of the data sources for conflicting data may offer alternative solutions.
- Although the CHERRY study has a relatively large number of participants, it is a regional cohort located in the developed area of China and as such, will not be nation-representative.

For peer review only

Introduction

Cardiovascular disease (CVD) becomes the leading global burden of diseases worldwide.[1] Epidemiological studies have suggested that the morbidity and mortality of CVD in developed countries remain stable over past decades. China's current cardiovascular epidemic is increasing with rapid economic development and changing lifestyles.[2] Some established researches, such as the Cardiovascular disease research using Linked Bespoke studies and Electronic health Records (CALIBER)[3] in the United Kingdom and the Cardiovascular Health in Ambulatory Care Research Team (CANHEART)[4] in Canada, showed that forging a partnership between the electronic health records (EHRs) and the population-based cohort studies for CVD epidemiology is becoming popular and useful. EHR-based data is enriched with individual-level longitudinal measurements of information. However, few existing studies in China successfully assemble cohorts with data collection based on EHRs at the population level so far. The success to link big data to population-based cohorts could promote advances both in improving cardiovascular outcomes and in facilitating healthcare services research.[5]

Several critical factors affect the quality of underlying data when EHRs were used for research, e.g. appropriate approaches to data management and linkages, operational definition for exposures, ascertainment and adjudication of outcomes, methods for missing data, relevant data analysis, and valid interpretation on clinical or public health integration and utility. These are all essential to generate robust findings.[6 7] Consequently, we aimed to describe the detailed methods and data sources used to establish a general population-based cohort in an eastern coastal area of China, i.e. The CHinese Electronic health Records Research in Yinzhou (CHERRY) study. By linking various data sets at the individual level using unique and encoded identifiers, the CHERRY study will be used as a research data platform to address several important research questions, including but not limited to the

For peer review only - http://bmjopen.bmj.com/site/about/guidelines.xhtml

following objectives:

1. What are the emerging risk factors, assembling both patient-level and community characteristics, to the incidence rates of major cardiovascular events in the developed area of China?
2. What is the appropriate CVD risk assessment model suitable for use in Chinese population due to nutrition transition?
3. How do the different screening strategies for targeting people at high risk of CVD perform in a real-world circumstance?
4. How does the regional health system deliver on various CVD prevention practices in terms of the improvements in cardiovascular outcomes?

Methods and analysis

The CHERRY study is a unique, population-based observational research resource, aimed at improving cardiovascular care and outcomes in China. A particular focus will be on cardiovascular risk prediction and population management, providing evidence to improve the primary prevention of cardiovascular events.

Study area

Yinzhou is a district of Ningbo in Zhejiang province (29°37′~29°57′ N, 121°08′~121°54′ E), located at 230 kilometers south of Shanghai in China with an area of 1,346 square kilometers (**Figure 1**). It has a total population of 1.24 million in 2016, 790,600 of which are permanent residents. This particular region, which is a developed area, was selected because of its outstanding general practice based healthcare units and unique integrated electronic health information system with primary care data (including important information on cardiovascular risk factors and their management in communities) in China. This enabled us to use the comprehensive big data for both epidemiological and clinical research in China.

For peer review only - http://bmjopen.bmj.com/site/about/guidelines.xhtml

Data sources and cohort establishment

Health information systems in Yinzhou included different administrative databases on general demographic characteristics, electronic medical records (EMRs), inpatient and outpatient claims, disease management, and death certificates. The special feature of this system in Yinzhou is that these databases are inherently linked to each other by individuals' unique and encoded identifier. The system had originally designed by the Yinzhou District Center for Disease Control and Prevention to facilitate routine primary care services for local general practitioners (GPs) since 2006. It was then gradually integrated with information on public health surveillance, population screening, disease management, and health care services. Since 2009, this regional system has nearly covered the entire health-related activities within this region of all people registered for health insurance, from birth to death, including children, adolescents, pregnant women, adults and the elderly. (Supplemental Material **Figure S1**)

Consequently, based on the data sources in this integrated system, the CHERRY study started in 2016, extracting individual participants' data within the system to establish a natural longitudinal population-based ambispective cohort study for cardiovascular care and outcomes research. The study will comprise all participants registered in the system if: (1) were above 18 years of age on 1st January 2009; (2) had complete information on date of birth, sex, and valid healthcare identifier; and (3) were Chinese nationality. We chose 1st January 2009 as the date of cohort inception to avoid the integration and preliminary test period of the system and to allow for the full coverage of the regional population. Once included in the CHERRY cohort, individuals remain in it until death or termination of the local health insurance (primarily due to moving out of the province). Follow-up is generally continuous in the health information system. CHERRY will update certain important information such as vital status, clinical outcomes and claims data for all cohort members annually from the administrative databases. A third party, i.e. Wonders

For peer review only - http://bmjopen.bmj.com/site/about/guidelines.xhtml

Information Co., Ltd., was engaged to perform the linkage and safe storage of the linked datasets, ensuring the privacy protection in CHERRY study. The description of CHERRY research cohort in relation to data sources in the administrative health information system is shown in **Figure 2** and **Table S1** (Supplemental Material). Each source captures a different aspect of the persons' life journey:

Socio-demographics

Basic demographic and socioeconomic information about residents stemmed from the population census and registered health insurance database in the health care system. Key data variables will include date of birth, sex, ethnic groups (e.g. Han, Muslim), marital status, education, occupation, and household information such as income, living space, etc.

Longitudinal measurements on cardiovascular risk factors

The local GPs in Yinzhou have built up an impressive frequency on general health checks for adults and regularity in conducting epidemiological surveys as part of routine primary care over ten years since China's Healthcare reform launched.[8] According to the New Rural Cooperative Medical Scheme (NRCMS) in China,[9] general health checks for adults are conducted once every two years by local GPs. CHERRY will include the longitudinal measurements on risk factors related to CVD at the individual level, e.g. smoking status, alcohol use, BMI and other obesity risk factors, and daily physical activity. Furthermore, screening of hypertension and diabetes mellitus in the general population ≥40 years is recommended by Chinese practice guidelines and has implemented in Yinzhou since 2009. Lipid is also frequently measured in the general population within the health check package paid by employers as staff welfare. Moreover, for adults who had a history of hypertension or diabetes, or individuals aged 60 years or over, a comprehensive medical examination is scheduled routinely at least once every year for disease management. In 2014, 348,571 adults had detailed records on this medical

For peer review only - http://bmjopen.bmj.com/site/about/guidelines.xhtml

examination within the health information system. Detailed information related to the CVD risk factors within the examination will be extracted to CHERRY, which included blood measurements on glucose, HbA1c, and lipid profiles (total cholesterol, HDL cholesterol, LDL cholesterol, triglycerides), as well as urine testing, etc. Besides, additional information from outpatient and inpatient EMRs will also be supplemented. For example, laboratory measurements of circulating markers of inflammation (such as homocysteine, C-reactive protein, albumin and the leucocyte count), or novel CVD related marker (e.g. NT-proBNP), or cardiovascular imaging information (e.g. progression of coronary artery calcium) will also be included. The core variables for CVD related factors and longitudinal measurements in the CHERRY study are listed in **Table 1**.

For peer review only

Health care services and medications

Outpatient and inpatient EMRs containing patients' health care services and medications information will be transferred to CHERRY. EMRs data involve nearly all physician visits, diagnostic tests, laboratory services, and medications for each individual, which is expected to rise over time. By the end of 2014, the EMRs had about 477,254 adult patients with the unique identifier from a network of over 289 primary care units and 5 hospitals across the Yinzhou District. The registered health insurance claims database will also be used, including cost related information on outpatient and inpatient admission in Yinzhou.

Clinical outcome events

For each individual, data will be sought on each of the following outcomes and on their dates of occurrence: non-fatal myocardial infarction; non-fatal CHD; non-fatal stroke; cause-specific mortality (or at least fatal CHD and fatal stroke) and other cardiovascular outcomes. Precise details of the diagnostic criteria used for the definition of cases have been sought from 2014 American College of Cardiology / American Heart Association Key Data Elements and Definitions for Cardiovascular

For peer review only - http://bmjopen.bmj.com/site/about/guidelines.xhtml

Endpoint Events in Clinical Trials.[10] Principal analyses will be based on events classified according to the 10th version of International Classification of Diseases (ICD-10) (**Table 2**). Attribution of death refers to the primary cause provided by cause-specific mortality from the death certificates in the health information system. Data undergo annual quality assessments. A description of the death certificates was reported previously.[11] In addition, incidence of CVD, hypertension, diabetes or cancer was also extracted from disease surveillance and management database where cases were required to be reported for disease management by local GPs once their diagnoses were confirmed. Diagnosis of these diseases made by the doctors in all regional hospitals will be automatically sent to the local GPs of patients in the system. Primary and secondary prevention patients were defined as those without (primary) or with (secondary) a known history of CVD (**Table 2**).

Environmental and ecological characteristics

For environmental exposure monitoring data, we will include exposures to major water and air pollutants from 8 environmental monitoring sites in Yinzhou, including heavy metal contamination and particles with aerodynamic diameter <2.5 μm ($PM_{2.5}$). Both have been associated with increased cardiovascular mortality.[12 13] In addition, various meteorological conditions such as air temperature and precipitation from all weather stations across Yinzhou during the study period will also be available. Previous studies in China have demonstrated that both short-term (in days) and longer-term (months or years) variations in temperature increase CVD morbidity and mortality.[14]

Sample size

Totally 965,349 Chinese adults aged over 18 years was registered in the health information system in 2009, and 22,501 deaths were observed from 1st January 2009 until 31st December 2014. According to sample size requirements for

For peer review only - http://bmjopen.bmj.com/site/about/guidelines.xhtml

prediction models,[15] for time to event outcomes, the number of participants experiencing the event must exceed 10 times the number of degrees of freedom, where the number of degrees of freedom includes the number of predictors screened for association with the outcome, all dummy variables, non-linear terms and interactions. Thus, the sample size is generally sufficient in the CHERRY study.

Data analysis plan

Detailed data analysis plan will follow the checklist in the Transparent reporting of a multivariable prediction model for individual prognosis or diagnosis (TRIPOD) guidelines[16] and recommendations by other studies.[15 17] In brief, data cleaning will proceed before examining exposure-outcome associations. Descriptive statistics will be used to determine values outside a plausible range, then outliers will be set to missing. Multiple imputation will be used to impute missing values on the predictors where appropriate. The CVD prediction models will be developed from sex-specific Cox proportional hazards models. The major CVD risk factors in established prediction models, such as the Framingham risk scores,[18] will be retained in our model directly. Then we will evaluate whether the predictive capability of the model will be improved by inclusion of additional predictors using measures of discrimination and reclassification. The clinical performance of the models will be assessed by discrimination *C* statistics, calibration chi-square and plots, net reclassification improvement (NRI) and the integrated discrimination improvement (IDI) index. Cross-validation will be used to evaluate internal consistency; when available, the prediction models will also be evaluated for external validation in other independent cohort studies, such as the Fangshan Cohort Study.[19] All statistical analysis will be conducted using the SAS system (version 9.4) and STATA software (version 14.1).

For peer review only - http://bmjopen.bmj.com/site/about/guidelines.xhtml

Discussion

The CHERRY study will be established to utilize longitudinal measurements of cardiovascular risk factors and disease prevention strategies in primary care among all permanent residences in Yinzhou. We aim to search for the integrated risk assessment tools for cardiovascular disease in Chinese population under the current economic level of development. We will also provide the evidence for different screening strategies in a real-world circumstance. We expect that these data will be useful to support a wide range of cardiovascular epidemiology and public health research. To our knowledge, the CHERRY study is among the first in China to establish the EHR-based research platform of linking big data across primary and secondary care and disease surveillance. In particular, it is unique in being able to trace a complete life journey in nearly a million adults in a general Chinese population. It used coded and inherently linked EHRs from primary care, hospitals, disease surveillance and, ultimately, death registries.

Linking big data from EHRs to population-based cohort might be powerful to investigate quality of care and to improve cardiovascular outcomes. On the one hand, epidemiological research using EHRs can help to quantify the need for clinical therapies, identify risk factors and suggest potential new drug targets. On the other hand, validated and updated risk prediction models may improve health decision-making by helping interventions to be targeted most appropriately and treated cost-effectively in different populations. Unfortunately, large-scale EHRs-based big data research on CVD from China, which has one-fifth of the world's total population, is currently underrepresented.[20]

Big data studies in developed countries are theoretically robust. For example, the CALIBER[3] in the United Kingdom was based the integrated health care database with both nationwide EHRs in primary care and ongoing national quality registries.

For peer review only - http://bmjopen.bmj.com/site/about/guidelines.xhtml

This may not be available in most countries. Sweden has similar health systems, while its primary care is organized regionally.[21] In addition, regional primary and ambulatory care data are also available for research linkages in the CANHEART study in Ontario, Canada.[4] Although cardiovascular disease registries, such as the Chinese National Stroke Registry (CNSR),[22] have been invaluable in collecting information about CVD and its treatment to drive research for secondary prevention in China, the evidence is often lacking for primary prevention in general populations. Fortunately, China's recent development in big data gathering efforts from the healthcare and medical sectors could facilitate the EHR-based epidemiological studies of CVD, especially in some developed areas. The CHERRY study has been inspired by all these studies.

It is known that less than 10 potentially modifiable risk factors might account for more than 90% of the population attributable risk of CVD in all regions of the world.[23 24] However, disparities for effects of individual risk factor on CVDs were also indicated across the different populations. Although the prevalence of CVDs decline in many developed countries with effective risk-lowering strategies to cardiovascular risk factors such as smoking cessation or salt reduction, the prevalence of CVDs in China is still increasing. The contemporary focus on CVD prevention by the latest guidelines emphasized the use of risk assessment to allow for appropriate prevention strategies to those at high risk of CVDs.[25] This is consistent with the objectives of the CHERRY study.

According to the checklist in the TRIPOD guidelines,[16] prediction models using real-world data should be developed to discriminate risk of death and events, and should be used in cost-effectiveness decision models for both public health and clinical practice. The CHERRY cohort will be established as a resource to encourage collaborative research based on individual participant data, in order to strengthen prognostic model development and external validation. For instance, recently

For peer review only - http://bmjopen.bmj.com/site/about/guidelines.xhtml

published China-PAR models[26] for CVD prediction in Chinese could be validated in the CHERRY cohort. Furthermore, large-scale EHR-based cohort studies with detailed longitudinal measurements on clinical risk and patient care data over time are warranted to better understand how risk factor influences the onset and progression of stroke and other CVDs. There are then great opportunities for the development of dynamic risk prediction models.[27] The CHERRY study can be leveraged to make risk prediction more organic, iterative, and contemporary.

The study has some limitations. Missing data will be one of the main limitations of any EHR-converted research platform in terms of data quality. Data completeness varies [e.g. 90.97% of people have at least one record on body mass index (BMI) measurement and 88.39% have their educational level in the system]. Developments in imputation within longitudinal cohorts may offer an alternative solution. Secondly, individual data may be conflicted among different sources of health system. For example, multiple records with similar but slightly different time of diagnosis of one subject may be available from different sources due to the varied timing accuracy. Priority of the sources in terms of the conflicting data will be set up. Events of one patient within certain range of time will be considered as a single event. The allowed time window is disease-specific. Finally, although the study has a relatively large number of subjects, it is a regional study which is located in the developed area of China. The population is therefore not nation-representative.

In summary, the CHERRY study has the potential to provide population-based insights into the quality and outcomes of cardiovascular care. CHERRY will serve to decrease the burden of obtaining data in a research-ready format and encourage research collaboration.

Ethics and dissemination

The CHERRY study has been approved by the Peking University Institutional Review

For peer review only - http://bmjopen.bmj.com/site/about/guidelines.xhtml

Board (IRB00001052-16011) and the local health authority. Results of the study will be disseminated through published journal articles, conferences and seminar presentations.

For peer review only

For peer review only - http://bmjopen.bmj.com/site/about/guidelines.xhtml

References

1. GBD 2016 Causes of Death Collaborators. Global, regional, and national age-sex specific mortality for 264 causes of death, 1980-2016: a systematic analysis for the Global Burden of Disease Study 2016. *Lancet* 2017;390(10100):1151-210.

2. Wu Y, Benjamin EJ, MacMahon S. Prevention and Control of Cardiovascular Disease in the Rapidly Changing Economy of China. *Circulation* 2016;133(24):2545-60.

3. Denaxas SC, George J, Herrett E, et al. Data resource profile: cardiovascular disease research using linked bespoke studies and electronic health records (CALIBER). *Int J Epidemiol* 2012;41(6):1625-38.

4. Tu JV, Chu A, Donovan LR, et al. The Cardiovascular Health in Ambulatory Care Research Team (CANHEART): using big data to measure and improve cardiovascular health and healthcare services. *Circ Cardiovasc Qual Outcomes* 2015;8(2):204-12.

5. Vasan RS, Benjamin EJ. The Future of Cardiovascular Epidemiology. *Circulation* 2016;133(25):2626-33.

6. Rumsfeld JS, Joynt KE, Maddox TM. Big data analytics to improve cardiovascular care: promise and challenges. *Nat Rev Cardiol* 2016;13(6):350-9.

7. Benchimol EI, Smeeth L, Guttmann A, et al. The REporting of studies Conducted using Observational Routinely-collected health Data (RECORD) statement. *PLoS Med* 2015;12(10):e1001885.

8. Blumenthal D, Hsiao W. Lessons from the East--China's rapidly evolving health care system. *N Engl J Med* 2015;372(14):1281-5.

9. Babiarz KS, Miller G, Yi H, et al. New evidence on the impact of China's New Rural Cooperative Medical Scheme and its implications for rural primary healthcare: multivariate difference-in-difference analysis. *BMJ* 2010;341:c5617.

For peer review only

10. Hicks KA, Tcheng JE, Bozkurt B, et al. 2014 ACC/AHA Key Data Elements and Definitions for Cardiovascular Endpoint Events in Clinical Trials: A Report of the American College of Cardiology/American Heart Association Task Force on Clinical Data Standards (Writing Committee to Develop Cardiovascular Endpoints Data Standards). *Circulation* 2015;132(4):302-61.

11. Wang JB, Gu MJ, Shen P, et al. Body Mass Index and Mortality: A 10-Year Prospective Study in China. *Sci Rep* 2016;6:31609.

12. Solenkova NV, Newman JD, Berger JS, et al. Metal pollutants and cardiovascular disease: mechanisms and consequences of exposure. *Am Heart J* 2014;168(6):812-22.

13. Zhang Y, Ji X, Ku T, et al. Heavy metals bound to fine particulate matter from northern China induce season-dependent health risks: A study based on myocardial toxicity. *Environ Pollut* 2016;216:380-90.

14. Chen R, Wang C, Meng X, et al. Both low and high temperature may increase the risk of stroke mortality. *Neurology* 2013;81(12):1064-70.

15. Taljaard M, Tuna M, Bennett C, et al. Cardiovascular Disease Population Risk Tool (CVDPoRT): predictive algorithm for assessing CVD risk in the community setting. A study protocol. *BMJ Open* 2014;4(10):e006701.

16. Collins GS, Reitsma JB, Altman DG, et al. Transparent reporting of a multivariable prediction model for individual prognosis or diagnosis (TRIPOD): the TRIPOD statement. *BMJ* 2015;350:g7594.

17. Woodward M, Tunstall-Pedoe H, Peters SA. Graphics and statistics for cardiology: clinical prediction rules. *Heart* 2017;103(7):538-45.

18. D'Agostino RB, Sr., Vasan RS, Pencina MJ, et al. General cardiovascular risk profile for use in primary care: the Framingham Heart Study. *Circulation* 2008;117(6):743-53.

19. Wu N, Tang X, Wu Y, et al. Cohort profile: the Fangshan Cohort Study of cardiovascular epidemiology in Beijing, China. *J Epidemiol* 2014;24(1):84-93.

For peer review only

20. Goldstein BA, Navar AM, Pencina MJ, et al. Opportunities and challenges in developing risk prediction models with electronic health records data: a systematic review. *J Am Med Inform Assoc* 2017;24(1):198-208.

21. Chung SC, Sundstrom J, Gale CP, et al. Comparison of hospital variation in acute myocardial infarction care and outcome between Sweden and United Kingdom: population based cohort study using nationwide clinical registries. *BMJ* 2015;351:h3913.

22. Wang Y, Cui L, Ji X, et al. The China National Stroke Registry for patients with acute cerebrovascular events: design, rationale, and baseline patient characteristics. *Int J Stroke* 2011;6(4):355-61.

23. Yusuf S, Hawken S, Ounpuu S, et al. Effect of potentially modifiable risk factors associated with myocardial infarction in 52 countries (the INTERHEART study): case-control study. *Lancet* 2004;364(9438):937-52.

24. O'Donnell MJ, Chin SL, Rangarajan S, et al. Global and regional effects of potentially modifiable risk factors associated with acute stroke in 32 countries (INTERSTROKE): a case-control study. *Lancet* 2016;388(10046):761-75.

25. Yang Q, Zhong Y, Gillespie C, et al. Assessing potential population impact of statin treatment for primary prevention of atherosclerotic cardiovascular diseases in the USA: population-based modelling study. *BMJ Open* 2017;7(1):e011684.

26. Yang X, Li J, Hu D, et al. Predicting the 10-Year Risks of Atherosclerotic Cardiovascular Disease in Chinese Population: The China-PAR Project (Prediction for ASCVD Risk in China). *Circulation* 2016;134(19):1430-40.

27. Jutte DP, Roos LL, Brownell MD. Administrative record linkage as a tool for public health research. *Annu Rev Public Health* 2011;32:91-108.

For peer review only - http://bmjopen.bmj.com/site/about/guidelines.xhtml

Acknowledgments

The authors thank the Health and Family Planning Bureau of Yinzhou District for providing access to the administrative databases used in the study.

Authors' contributions

HL, XT and PG drafted the manuscript. HL, XT, PS and PG conceived of and designed the study. DZ, JW, JZ, PL and YS made substantial contributions to study design. HL, JW and PS are responsible for study coordination; XT, PS and JZ are responsible for data quality control; DZ, PL and YS are responsible for data wrangling; XT, DZ, YS and PG are responsible for data analysis. All authors contributed to the writing of the study protocol in an iterative manner, and have read and approved the final manuscript.

Funding

This study is supported by the National Natural Science Foundation of China (91546120, 81573226), and the National Thousand Talents Program for Distinguished Young Scholars, China (QNQR201501).

Competing interests

The authors declare that they have no conflicts of interests.

For peer review only

For peer review only - http://bmjopen.bmj.com/site/about/guidelines.xhtml

Figure legends

Figure 1 Study location for the CHinese Electronic Health Records Research in Yinzhou (CHERRY)

Figure 2 Data sources to create the CHinese Electronic Health Records Research in Yinzhou (CHERRY) cohort

Notes: Although the main purpose of the CHERRY study is to focus on adults, we could also have some data sources for infants, children, and pregnant women in the health information system to identify other potential influence of early life exposures on cardiovascular morbidity and mortality; for example the birth weight from the birth certificate system or some maternal exposures from the antenatal examinations.

For peer review only

Tables

Table 1 List of core risk factors for CVD in the CHERRY study

**Characteristics of first registration**
A unique but encoded identifier
Date of entry of first registration
Date of birth, sex and ethnic groups
Marital status, education, occupation and socio-economic status (household income, living space, etc.)
Smoking and alcohol use (current/former/never; amount/duration etc.)
Physical activity
Weight, height, waist and hip circumference
History of hypertension and diabetes mellitus
Prior history of coronary heart disease (in particular myocardial infarction and angina), stroke, transient ischemic attack (TIA), and peripheral vascular disease (PVD)
**Longitudinal measurements on risk factors**
Date of the visit
Smoking and alcohol use (current/former/never; amount/duration etc.)
Physical activity
Weight, height, waist and hip circumference
Diagnosis of hypertension and diabetes mellitus
Systolic and diastolic blood pressure
Metabolic factors (including fasting glucose, post-load glucose, and glycosylated hemoglobin)
Lipid profiles: total, high- and low-density lipoprotein cholesterol; triglycerides (including information about fasting status at time blood samples were taken);
Blood urea nitrogen, creatinine, uric acid
**Inpatient and Outpatient Electronic medical records**
Date of the record
Use of cardiovascular medications (including anti-hypertensive drugs, 'statins',

For peer review only - http://bmjopen.bmj.com/site/about/guidelines.xhtml

fibrates) and other medications (e.g., hypoglycemic agents, hormone replacement therapy)
Lipid profiles: total, high- and low-density lipoprotein cholesterol; triglycerides, lipoprotein (a); apolipoprotein-AI and -B;
Inflammatory markers (including homocysteine, C-reactive protein, fibrinogen, albumin, interleukin-6 and the leucocyte count)
Haemostatic factors (including von-Willebrand factor and fibrin D-dimer)
Novel CVD related markers (e.g. NT-proBNP)
Cardiovascular imaging information (e.g. progression of coronary artery calcium, carotid intima-media thickness)
Blood urea nitrogen, albumin, uric acid, creatinine, and Cystatin C
Urine Albumin-to-Creatinine Ratio (UACR)
Principal diagnosis of outpatient or inpatient admission
Cost of outpatient or inpatient admission (including fees for diagnosis, prescription, laboratory test, surgery etc.)
**Environmental and ecological data**
Date and region of surveillance
Air temperature and precipitation
Particles with aerodynamic diameter <2.5 μm ($PM_{2.5}$)
Heavy metal concentration in water (lead, cadmium, mercury and arsenic)

For peer review only

For peer review only - http://bmjopen.bmj.com/site/about/guidelines.xhtml

Table 2 Definitions of major outcomes in the CHERRY study

Events of interest	ICD-10 Code
**Primary events of interest**	
Death due to	
Ischemic heart disease	I20-I25
Cerebrovascular disease	I60-69
Major cardiovascular disease	I00-I78
All-cause mortality	
Hospitalization with main diagnosis of	
Myocardial infarction	I21, I22
Stroke	I60, I61, I63 (excluding I63.6), I64, H34.1
Congestive heart failure	I50
Outpatient visit with main diagnosis of	
Hypertension*	I10, I11, I12, I13, I15
Diabetes mellitus†	E10, E11, E13, E14
Cardiovascular disease	I00-I99
**Secondary events of interest (fatal and non-fatal)**	
ST-Elevation Myocardial Infarction (STEMI)	R94.30
Non-ST segment elevation myocardial infarction (NSTEMI)	R94.31
Ischemic stroke	I63, I64, H34.1 (excluding I63.6)
Hemorrhagic stroke	I60, I61
Transient ischemic attack	G45 (excluding G45.4), H34.0
Atrial fibrillation	I48
Aortic aneurysm / aortic dissection	I71
Peripheral artery disease	I70.2, I73.9, I74.3, I74.4

* Hypertension includes either registered in the hypertension management system or self-reported history of hypertension during health checks or one hospital admission with a hypertension diagnosis.

† Diabetes mellitus includes either registered in the diabetes management system or self-reported history of diabetes during health checks or one hospital admission with a diabetes diagnosis.

For peer review only - http://bmjopen.bmj.com/site/about/guidelines.xhtml

Supplemental Materials

Figure S1. Administrative databases for CHERRY cohort

Note: The green ones in the databases are currently not included in the CHERRY cohort.

Table S1. Description of CHERRY study data sources and dataset codes

For peer review only

For peer review only - http://bmjopen.bmj.com/site/about/guidelines.xhtml

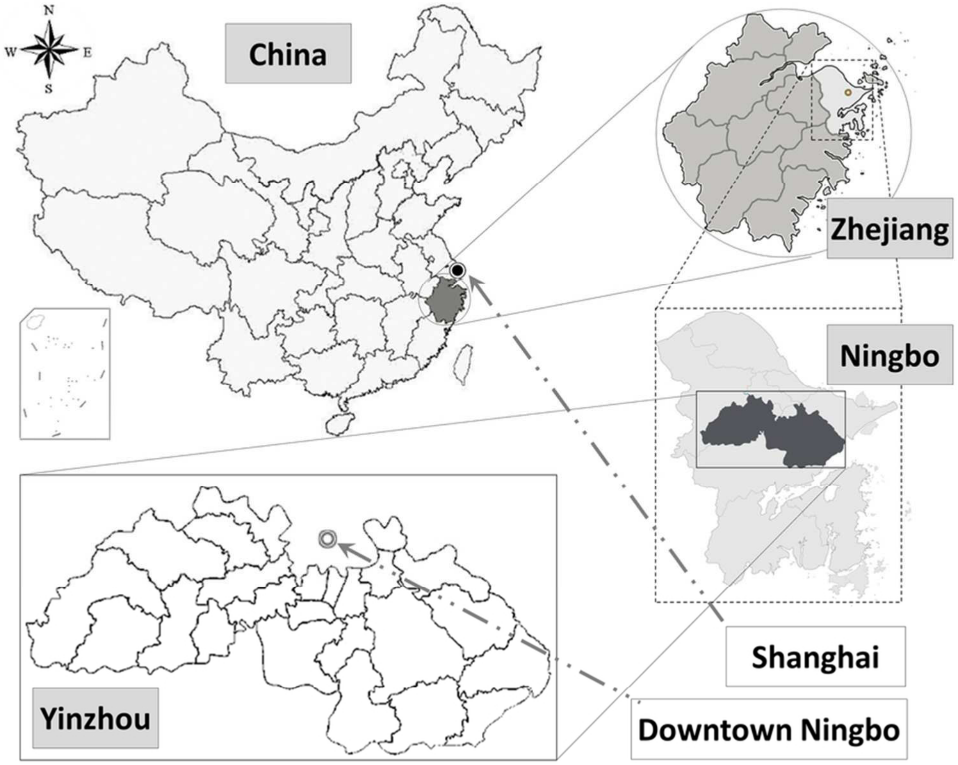

Study location for the CHinese Electronic Health Records Research in Yinzhou (CHERRY)

80x64mm (300 x 300 DPI)

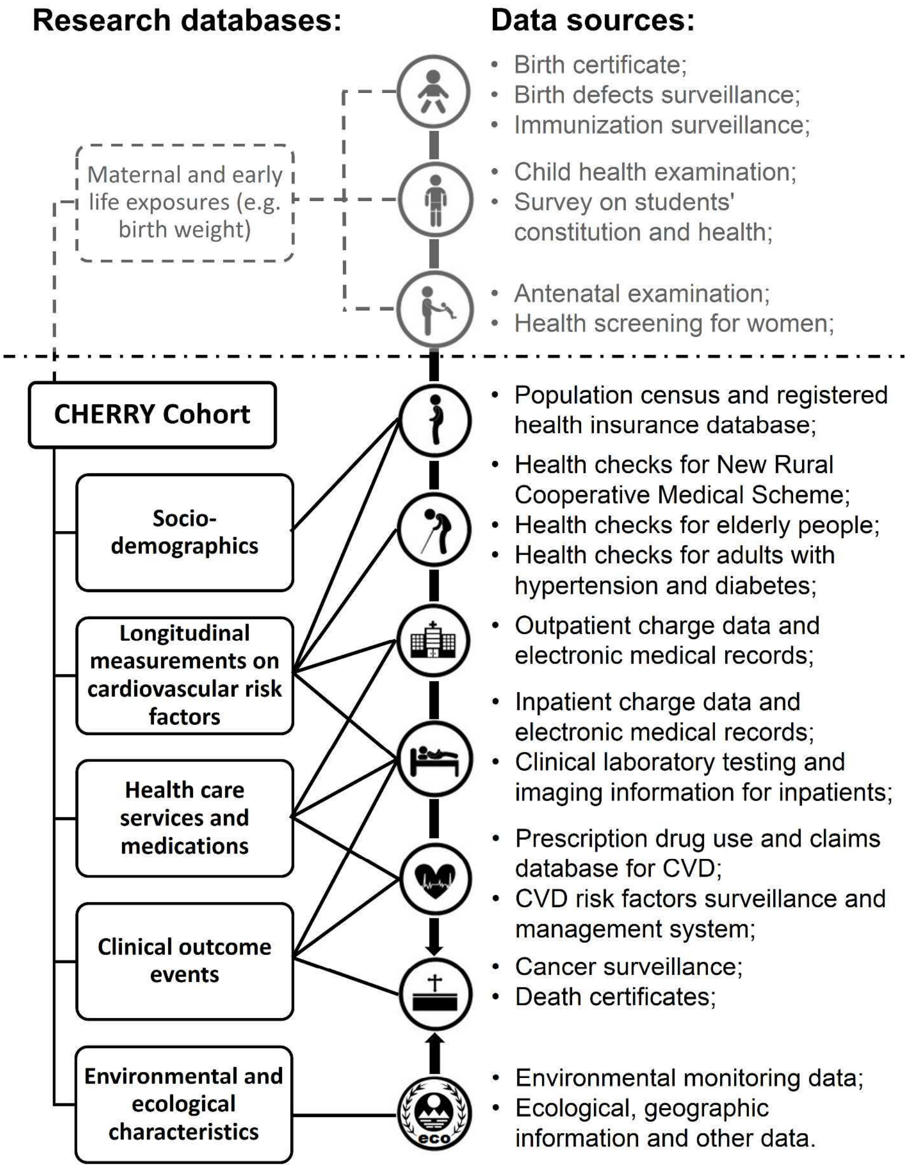

Data sources to create the CHinese Electronic Health Records Research in Yinzhou (CHERRY) cohort
Notes: Although the main purpose of the CHERRY study is to focus on adults, we could also have some data sources for infants, children, and pregnant women in the health information system to identify other potential influence of early life exposures on cardiovascular morbidity and mortality; for example the birth weight from the birth certificate system or some maternal exposures from the antenatal examinations.

900x1200mm (96 x 96 DPI)

Supplemental Material

Table S1. Description of CHERRY study data sources and dataset codes

Research databases	Data sources	Dataset codes	Description
Socio-demographics	Registered health insurance database	KTBASE_PERSON	Registered person insurance records
		KTBASE_CARD	Registered person insurance card information
		KTHRA_HEALTH_ARCHIVES	Registered person health records
		KTHRA_HEALTH_AIDINFO	Registered person supplementary health records
		KTHRA_PRACTICE	Life-style risk factors information records
		KTHRA_FAMILY_HISTORY	Family history records
		KTHRA_DIAGNOSES	Disease history records
	Population census database	KTHRA_HOME	Registered household records
		KTHRA_HOME_PROBLEMS	Major household information records

For peer review only - http://bmjopen.bmj.com/site/about/guidelines.xhtml

Longitudinal measurements on cardiovascular risk factors	Health checks for New Rural Cooperative Medical Scheme (NRCMS) database	KTPEIS_S401_1	Health checks records
		KTPEIS_S401_2	Detailed health checks records
		KTPEIS_S401_3	Health checks summary
		KTPEIS_MAN_CHECK	Adult health checks registration information
		KTPEIS_MAN_LIFESTYLE	Adult life-style information records
		KTPEIS_MAN_CHECKHEALTH	Adult physical examination records
		KTPEIS_MAN_AIDCHECK	Adult laboratory testing records
		KTPEIS_MAN_MAINPROBLEM	Adult major health problem records
		KTPEIS_MAN_INHOS	Adult inpatient hospital admission records
		KTPEIS_MAN_MAIN_DRUG	Adult current medication information
		KTPEIS_MAN_ASSESSMENT	Adult health assessment information

For peer review only

For peer review only - http://bmjopen.bmj.com/site/about/guidelines.xhtml

	Health checks for elderly people database	KTHRA_ELDER_VISITS	Elderly people follow-up records
		KTHRA_ELDER_ARCHIVES	Elderly people health assessment information
	Health checks for adults with hypertension database	KTCDMS_CARD	Adults with hypertension registration information
		KTCDMS_HT_CARD	Detailed information on adults with hypertension records
		KTCDMS_HT_CARD_CHECK	Health checks records for adults with hypertension
		KTCDMS_HT_VISITS	Follow-up records for adults with hypertension
		KTCDMS_HT_VISITS_DETAIL	Detailed follow-up records for adults with hypertension
		KTCDMS_HT_MEDIDETAIL	Medication records for adults with hypertension
		KTCDMS_HT_YEAREVAL	Annual health assessment for adults with hypertension
	Health checks for adults with diabetes database	KTCDMS_CARD	Adults with diabetes registration information
		KTCDMS_DM_CARD	Detailed information on adults with diabetes records

For peer review only - http://bmjopen.bmj.com/site/about/guidelines.xhtml

	KTCDMS_DM_VISITS	Follow-up records for adults with diabetes
	KTCDMS_DM_VISITS_DETAIL	Detailed follow-up records for adults with diabetes
	KTCDMS_DM_MEDIDETAIL	Medication records for adults with diabetes
	KTCDMS_DM_YEAREVAL	Annual health assessment for adults with diabetes
Cardiovascular high-risk population	KTCDMS_HIGHRISK_CARD	Cardiovascular high-risk person registration chart
	KTCDMS_HIGHRISK_VISITS	Cardiovascular high-risk person follow-up records
	KTCDMS_HIGHRISK_DETAIL	Detailed cardiovascular high-risk person follow-up records
Clinical examination records database	KTEXAMINE_REPORT_INFO	Clinical examination and imaging information records
Clinical laboratory testing database	KTASSAY_REPORT_INFO	Laboratory testing records
	KTASSAY_REPORT_DETAIL	Detailed laboratory testing records

For peer review only
For peer review only - http://bmjopen.bmj.com/site/about/guidelines.xhtml

Health care services and medications	Outpatient medical records database	KTOUT_REGINFO	Outpatient registration information records
		KTDOC_DIAGNOSE	Outpatient diagnosis records
	Outpatient prescription database	KTDOC_PRESCRIPTION	Outpatient prescription records
		KTDOC_PRESCRIPTION_LIST	Detailed outpatient prescription records
		KTDOC_EXPENSES	Outpatient treatment expenses records
	Outpatient charge database	KTOUT_CHARGE	Outpatient charge records
		KTOUT_CHARGEDETAIL	Detailed outpatient charge records
	Inpatient medical records database	KTINHOS_REGISTER	Inpatient registration information records
		KTINHOS_PATIENT_HOME	Inpatient medical records cover sheet information
		KTINHOS_OUTBRIEF	Discharge summary records

For peer review only - http://bmjopen.bmj.com/site/about/guidelines.xhtml

	Inpatient prescription database	KTINHOS_MEDICAL_ORDER	Inpatient treatment records
		KTINHOS_DIAGNOSIS	Inpatient diagnosis records
		KTINHOS_SURGERY	Inpatient surgery records
	Inpatient charge database	KTINHOS_CHARGE	Inpatient charge records
		KTINHOS_CHARGE_OUT	Discharge bill records
		KTINHOS_CHARGE_OUT_DETAIL	Detailed discharge bill records
Clinical outcome events	Cardiovascular diseases surveillance and management system	KTCDMS_CVA_CARD	Detailed information on adults with cardiovascular disease records
		KTCDMS_CVA_VISITS	Follow-up records for adults with cardiovascular diseases
		KTCDMS_CVA_VISITS_DETAIL	Detailed follow-up records for adults with cardiovascular diseases
		KTCDMS_CVA_MEDIDETAIL	Medication records for adults with cardiovascular diseases

For peer review only - http://bmjopen.bmj.com/site/about/guidelines.xhtml

Cancer surveillance database	KTCDMS_TUMOR_CARD	Detailed information on adults with cancer records
	KTCDMS_TUMOR_VISITS	Follow-up records for adults with cancer
	KTCDMS_TUMOR_VISITS_DETAIL	Detailed follow-up records for adults with cancer
	KTCDMS_TUMOR_MEDIDETAIL	Medication records for adults with cancer
Database of death records	KTHRA_DEATH_RECORD	Death certificates

For peer review only - http://bmjopen.bmj.com/site/about/guidelines.xhtml

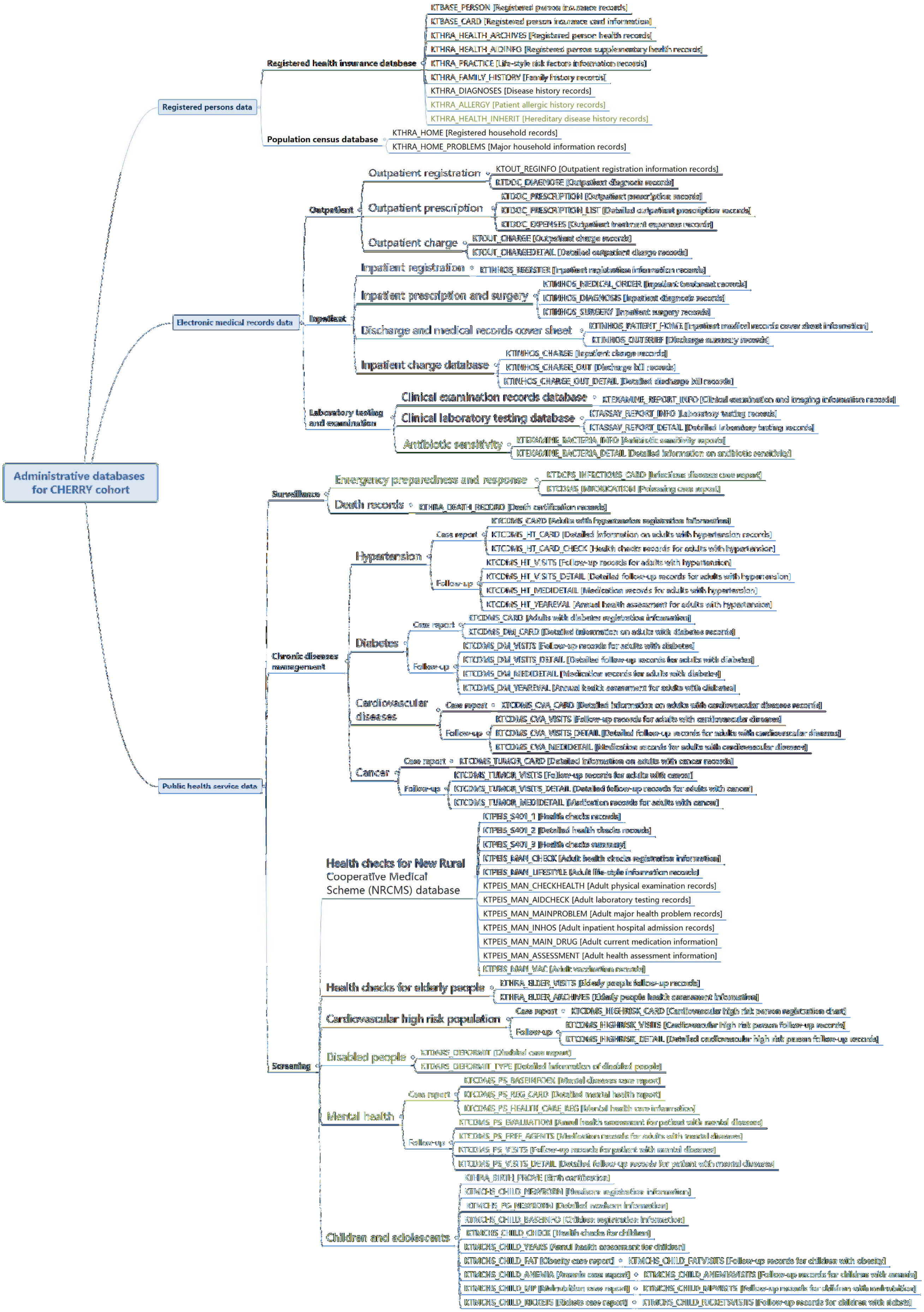

Figure S1. Administrative databases for CHERRY cohort

BMJ Open

Using big data to improve cardiovascular care and outcomes in China: a protocol for the CHinese Electronic health Records Research in Yinzhou (CHERRY) study

Journal:	*BMJ Open*
Manuscript ID	bmjopen-2017-019698.R1
Article Type:	Protocol
Date Submitted by the Author:	30-Nov-2017
Complete List of Authors:	Lin, Hongbo; Yinzhou District Center for Disease Control and Prevention<br>TANG, Xun; Peking University Health Science Center, Department of Epidemiology and Biostatistics<br>Shen, Peng; Yinzhou District Center for Disease Control and Prevention<br>Zhang, Dudan; Peking University Health Science Center, Department of Epidemiology and Biostatistics<br>Wu, Jinguo; Wonders Information Co., Ltd.<br>Zhang, Jingyi; Wonders Information Co., Ltd.<br>Lu, Ping; Wonders Information Co., Ltd.<br>Si, Yaqin; Peking University Health Science Center, Department of Epidemiology and Biostatistics<br>GAO, Pei; Peking University Health Science Center, Department of Epidemiology and Biostatistics
**Primary Subject Heading**:	Cardiovascular medicine
Secondary Subject Heading:	Epidemiology
Keywords:	cardiovascular diseases, electronic health records, Chinese

SCHOLARONE™
Manuscripts

Using big data to improve cardiovascular care and outcomes in China: a protocol for the CHinese Electronic health Records Research in Yinzhou (CHERRY) study

Hongbo Lin, [1]* Xun Tang, [2]* Peng Shen, [1] Dudan Zhang, [2] Jinguo Wu, [3] Jingyi Zhang, [3] Ping Lu, [3] Yaqin Si, [2] Pei Gao, [2#]

[1] Yinzhou District Center for Disease Control and Prevention, Ningbo 315100, China

[2] Department of Epidemiology and Biostatistics, Peking University Health Science Center, Beijing 100191, China

[3] Wonders Information Co., Ltd., Shanghai 201112, China

* Drs. Hongbo Lin and Xun Tang contributed equally to this paper.

[#] **Correspondence to:**

Prof. Pei Gao, PhD

Department of Epidemiology and Biostatistics,

Peking University Health Science Center,

38 Xueyuan Road, Beijing 100191, China

Tel/Fax: +86-10-82805642

Email: peigao@bjmu.edu.cn

Total word count: 5020 words, Abstract: 298 words, 2 Figures and 3 Tables, 33 References, 3 Supplementary files

For peer review only

For peer review only - http://bmjopen.bmj.com/site/about/guidelines.xhtml

Abstract

Introduction: Data based on electronic health records (EHRs) is rich with individual-level longitudinal measurement information and are becoming an increasingly common data source for clinical risk prediction worldwide. However, few EHR-based cohort studies are available in China. Harnessing EHRs for research requires a full understanding of data linkages, management, and data quality in large data sets, which presents unique analytic opportunities and challenges. The purpose of this study is to provide a framework to establish a uniquely integrated EHR database in China for scientific research.

Methods and Analysis: The CHinese Electronic health Records Research in Yinzhou (CHERRY) study will extract individual participant data within the regional health information system of an eastern coastal area of China to establish a longitudinal population-based ambispective cohort study for cardiovascular care and outcomes research. A total 1,053,565 Chinese adults aged over 18 years were registered in the health information system in 2009, and there were 23,394 deaths from 1 January 2009 to 31 December 2015. The study will include information from multiple epidemiological surveys; EHRs for chronic disease management; and health administrative, clinical, laboratory, drug, and electronic medical record databases. Follow-up of fatal and non-fatal clinical events is achieved through records linkage to the regional system of disease surveillance, chronic disease management and electronic medical records (based on diagnostic codes from the International Classification of Diseases [the Tenth Revision]). The CHERRY study will provide a unique platform and serve as a valuable big data resource for cardiovascular risk prediction and population management, for primary and secondary prevention of cardiovascular events in China.

Ethics and dissemination: The CHERRY study was approved by the Peking

For peer review only

University Institutional Review Board (IRB00001052-16011) in April 2016. Results of the study will be disseminated through published journal articles, conferences and seminar presentations, and on the study website (http://www.cherry-study.org).

For peer review only

Strengths and limitations of this study

- The Chinese Electronic health Records Research in Yinzhou (CHERRY) study is a large, natural population-based, observational cohort study linking big data of integrated individual-level electronic health records (EHRs).
- The CHERRY study is among the first in China to establish a research platform from EHRs system for investigating a wide range of important issues regarding primary and secondary prevention of cardiovascular disease in a real-world circumstance.
- The CHERRY study is unique in its ability to trace the complete lifetime health care journey using birth certificates, health checks, primary care visits, hospitalizations, disease surveillance, and ultimately, death certificates for one million adults in the general Chinese population.
- Missing data and conflicting data might be the main limitations of any EHR-converted big data research in terms of data quality; however, imputation for missing data within longitudinal cohorts and setup of the priority of data sources for conflicting data may offer alternative solutions.
- Although the CHERRY study has a relatively large number of participants, it is a regional cohort located in a developed area of China and as such, will not be nationally representative.

For peer review only

Introduction

Cardiovascular disease (CVD) is the leading global disease burden worldwide.[1] Epidemiological studies have suggested that age-standardized CVD mortality rates have been falling in developed countries in recent decades.[2] However, China's current cardiovascular epidemic is increasing with rapid economic development and changing lifestyles.[3] Some established studies, such as the Cardiovascular disease research using Linked Bespoke studies and Electronic health Records (CALIBER)[4] in the United Kingdom and Cardiovascular Health in Ambulatory Care Research Team (CANHEART)[5] in Canada, have showed that forging a partnership between electronic health records (EHRs) and population-based cohort studies for CVD epidemiology is useful and growing. EHR-based data is rich with individual-level longitudinal measurement information. However, few existing studies in China to date have successfully assembled cohort study based on EHRs at the population level. Success in linking big data to population-based cohort could promote advances both in improving cardiovascular outcomes and facilitating health care services research.[6]

Several critical factors affect the quality of EHR-based data for research, e.g., appropriate approaches to data management and linkages, operational definitions for exposures, ascertainment and adjudication of outcomes, methods for handling missing data, and valid interpretation of clinical or public health integration and utility. These are all essential to generating robust findings.[7 8] Consequently, in this protocol, we aimed to describe the detailed methods and data sources used to establish an EHR-based general population-based cohort study in an eastern coastal area of China, the CHinese Electronic health Records Research in Yinzhou (CHERRY) study. By linking various data sets at the individual level using unique and encoded identifiers, the CHERRY study will be used as a research platform to address several important research questions, including but not limited to the following objectives:

For peer review only - http://bmjopen.bmj.com/site/about/guidelines.xhtml

1. What are the emerging risk factors, assembling both individual-level and community-level characteristics, for the incidence of major cardiovascular events in this developed area of China?
2. What is the up-to-date and suitable CVD risk assessment model to use for Chinese population in nutrition transition?
3. How do the different screening strategies for targeting people at high risk of CVD perform in a real-world circumstance?
4. How does the regional health system deliver various CVD prevention practices in terms of cardiovascular outcome improvement?

Methods and analysis

The CHERRY study is a unique, population-based observational research resource that is aimed at improving cardiovascular care and outcomes in China. A particular focus will be on cardiovascular risk prediction and population management, providing evidence to improve the primary and secondary prevention of cardiovascular events.

Study area

Yinzhou is a district of Ningbo in Zhejiang province (29°37′-29°57′ N, 121°08′-121°54′ E), located 230 kilometers south of Shanghai, China, with an area of 1,346 km^2 (**Figure 1**). Yinzhou had a total population of 1.24 million in 2016, including 790,600 permanent residents. This particular region is a developed area and was selected because of its outstanding general practice based primary care units and unique integrated electronic health information system. Particularly, the system has captured rich information from primary care units, including important information on cardiovascular risk factors and their disease management among communities. This enables us to use comprehensive big data for both

For peer review only - http://bmjopen.bmj.com/site/about/guidelines.xhtml
For peer review only

epidemiological and clinical research in China.

Data sources and cohort establishment

Health information systems in Yinzhou include different administrative databases of general demographic characteristics, health check information, inpatient and outpatient electronic medical records (EMRs), health insurance database, disease management, and death certificates, etc. The special feature of the system in Yinzhou is that these databases are inherently linked to each other by a unique and encoded identifier for each individual. The system was originally designed in 2006 by the Yinzhou District Center for Disease Control and Prevention to facilitate routine primary care services for local general practitioners (GPs). It was then gradually integrated with information on public health surveillance, population screening, disease management, health information system in hospitals and other healthcare services. Since 2009, this regional system has covered nearly all health-related activities of residents within this region, from birth to death, including children, adolescents, pregnant women, adults and elderly people (**Figure 2** and **Figure S1**). Now 98% of permanent residents in Yinzhou have registered in the health information system with a valid healthcare identifier.

Consequently, based on the data sources in this integrated system, the CHERRY study started in 2016 and is now extracting individual participants' data within the system to establish a natural, longitudinal, population-based, ambispective cohort study for cardiovascular care and outcomes research. The study comprises all participants registered in the system if they: (1) were over 18 years of age on 1 January 2009; (2) have complete information on date of birth, sex, and a valid healthcare identifier; (3) have been living in Yinzhou for at least 6 months; and (4) are Chinese nationality. We chose 1 January 2009 as the date of cohort inception to bypass the integration and preliminary test period of the system and to allow for full coverage of the regional population. Once included in the CHERRY cohort,

For peer review only - http://bmjopen.bmj.com/site/about/guidelines.xhtml

individuals remain in it until death or termination of local health insurance (primarily owing to moving out of the province). Follow-up is generally continuous in the health information system. CHERRY will update certain important information such as vital status, clinical outcomes, and claims data for all cohort members annually from the administrative databases. A third party, Wonders Information Co., Ltd., was engaged to handle linkage and safe storage of the linked datasets, ensuring privacy protection in the CHERRY study. A description of the CHERRY research cohort in relation to data sources in the administrative health information system is shown in **Figure 2** and **Table S1**. Each source captures a different aspect of a person's lifetime health care journey, as follows.

Socio-demographics

Basic demographic and socioeconomic information of residents stems from the population census and registered health insurance database in the health information system. Key data variables will include date of birth, sex, ethnic group (e.g., Han, Muslim), marital status, education, occupation, and household information such as income, living space, etc.

Longitudinal measurement of cardiovascular risk factors

Local GPs in Yinzhou have built up an impressive scheme on frequent health checks among adults and regular epidemiological surveys as part of primary care routine services over the 10 years after China's healthcare reform was initially launched.[9] According to the New Rural Cooperative Medical Scheme (NRCMS) in China,[10] general health checks for adults in rural areas are conducted once every 2 years by local GPs. CHERRY then includes longitudinal measurements of risk factors related to CVD at the individual level, e.g., smoking status, alcohol use, BMI and other obesity risk factors, and daily physical activity. Furthermore, screening of hypertension and diabetes mellitus in the general population aged 40 years and older is recommended by Chinese clinical practice guidelines and has been

For peer review only - http://bmjopen.bmj.com/site/about/guidelines.xhtml

implemented in Yinzhou since 2009. Especially, lipid levels are frequently measured for the general population within the health check package paid for by employers, as part of employee welfare. Moreover, for adults with a history of hypertension or diabetes or individuals aged 60 years or over, a comprehensive medical examination is scheduled routinely at least once every year, for disease management. In total, 53% of individuals aged 40 years or over had at least one general health check information (e.g., blood pressure measurements) within the system. Detailed information related to CVD risk factors within the examination is extracted to CHERRY, which includes blood measurements on glucose, Hemoglobin A1c (HbA1c), and lipid profiles (total cholesterol, HDL cholesterol, LDL cholesterol, triglycerides) as well as urine testing, and so on. Additional information from outpatient and inpatient EMRs will also be supplemented. Laboratory measurements in EMRs of circulating inflammation markers (e.g., homocysteine, C-reactive protein, albumin, and leucocyte count), novel CVD related markers [e.g., N-terminal pro b-type natriuretic peptide (NT-proBNP)], or cardiovascular imaging information (e.g., progression of coronary artery calcium) are included when available. The core variables for CVD-related factors and longitudinal measurements in the CHERRY study are listed in **Table 1**.

Health care services and medications

Outpatient and inpatient EMRs containing information of patients' health care services and medications will be transferred to CHERRY. EMR data contains physician visits, primary and secondary disease diagnosis, laboratory services, medications (indication, strength and dosage instructions) and so on from a network of 5 hospitals and over 289 primary care units across Yinzhou. The system (and CHERRY study) contained EMRs in all the hospitals (both public and private hospitals) and primary care units within Yinzhou but no pharmacy stores. Therefore, the dispensation of medicine from both hospitals and primary care units are available (prescription medicines are always taken directly from the pharmacy

For peer review only - http://bmjopen.bmj.com/site/about/guidelines.xhtml

within the hospitals/primary care units in China). Both individuals with and without health insurance can access the primary care and hospital services and therefore are all included in the system/study. By the end of 2015, 95.9% (1,010,658/1,053,565) adult participants with the unique identifier had EMRs in the system, receiving at least one clinical service (hospital or primary care). For patients receiving care outside Yinzhou (e.g., patients might go to famous hospitals in Shanghai for certain complex surgical procedures), major non-fatal events occurred (e.g., CVD and cancer, etc) are tracked from both disease surveillance and chronic disease management system. In this case, patients generally reported to local hospital/GPs for after-surgery health check services and drug prescription. Fatal events are tracked from death registry where death certificates issued from hospitals outside Yinzhou are available. For the information related to the primary disease diagnosis and medication used in hospitals outside the region, limited information is also extracted from the health insurance database. In Yinzhou, 95.7% of permanent residents are covered by the national health insurance. Residents of all ages were covered by the system. However, only adults above 18 years of age on 1 January 2009 are included in the CHERRY study.

Clinical outcome events

For each individual, data will be sought on each of the following outcomes and their dates of occurrence: non-fatal myocardial infarction, non-fatal coronary heart disease (CHD), non-fatal stroke, cause-specific mortality (or at least fatal CHD and fatal stroke), and other cardiovascular outcomes. Precise details of the diagnostic criteria used to define cases have been sought from the 2014 American College of Cardiology/American Heart Association Key Data Elements and Definitions for Cardiovascular Endpoint Events in Clinical Trials.[11] Principal analyses will be based on events classified according to the International Classification of Diseases, Tenth Revision (ICD-10) (**Table 2**). In the CHERRY study, for fatal outcomes, attribution of death refers to the primary cause provided by cause-specific mortality on death

For peer review only - http://bmjopen.bmj.com/site/about/guidelines.xhtml

certificates in the health information system. Data undergo annual quality assessments. A description of the death certificates has been reported previously.[12] For non-fatal outcomes, multiple sources exist in the system for the outcome definition, i.e., disease management database (primary care), EMRs database (hospital care), health insurance database, and disease surveillance database (disease registry). We define the disease surveillance database as gold standard. In Yinzhou, CVD, hypertension, diabetes, or cancer cases were required to be reported for disease surveillance and management by local GPs once the diagnoses were confirmed. Diagnosis of these diseases made by the physicians in all regional hospitals will be automatically sent to the local GPs of patients in the system. Criteria used for the diagnosis of incident cardiovascular morbidity in each source were described in **Table S2**. We define a "definite" event if two or more sources excluding health insurance database reported as a case. A "probable" event is defined if any source (including health insurance database) reported as a case. Cross-validation will be further investigated to improve the data quality and diagnostic validity. Primary and secondary prevention patients are defined as those without (primary) or with (secondary) a known history of CVD (**Table 2**).

Environmental and ecological characteristics

For environmental exposure monitoring data, we will include exposures to major water and air pollutants from eight environmental monitoring sites in Yinzhou, including heavy metal contamination and particles with aerodynamic diameter <2.5 μm ($PM_{2.5}$). These have been associated with increased cardiovascular mortality.[13] [14] In addition, various meteorological conditions, such as air temperature and precipitation, from all weather stations across Yinzhou during the study period will also be available. Previous studies in China have demonstrated that both short-term (days) and longer-term (months or years) variations in temperature increase CVD morbidity and mortality.[15]

For peer review only - http://bmjopen.bmj.com/site/about/guidelines.xhtml

Sample size

A total of 1,053,565 Chinese adults aged over 18 years were registered in the health information system. According to sample size requirements for prediction models,[16] for time to event outcomes, the number of participants experiencing the event must exceed 10 times the number of degrees of freedom, where the number of degrees of freedom includes the number of predictors screened for association with the outcome, all dummy variables, non-linear terms and interactions. By the end of 2015, there were 23,394 deaths (12,669 men and 10,725 women) and 14,217 cardiovascular events (7,825 men and 6,392 women) from 1 January 2009 to 31 December 2015. Thus, the sample size is generally sufficient for the CHERRY study.

Data analysis plan

A detailed data analysis plan will follow the checklist in the Transparent reporting of a multivariable prediction model for individual prognosis or diagnosis (TRIPOD) guidelines[17] and recommendations by other studies.[16 18] In brief, data cleaning will proceed before examining exposure-outcome associations. Descriptive statistics will be used to determine values outside a plausible range, then outliers will be set to missing. Multiple imputation will be used to impute missing values for the predictors, where appropriate. CVD prediction models will be developed from sex-specific Cox proportional hazards models. The main CVD risk factors in established prediction models, such as Framingham risk scores,[19] will be retained in our model directly. We will then evaluate whether the predictive capability of the model will be improved by inclusion of additional predictors, using measures of discrimination and reclassification. The clinical performance of the models will be assessed by discrimination *C* statistics, calibration chi-square and plots, net reclassification improvement (NRI) and the integrated discrimination improvement (IDI) index. Cross-validation will be used to evaluate internal consistency; when available, the prediction models will also be evaluated for external validation in

For peer review only - http://bmjopen.bmj.com/site/about/guidelines.xhtml

other independent cohort studies, such as the Fangshan Cohort Study.[20] All statistical analysis will be conducted using the SAS system, version 9.4 (SAS Institute, Cary, NC, USA) and STATA software, version 14.1 (StataCorp LLC, College Station, TX, USA).

Discussion

The CHERRY study is established to use longitudinal measurements of cardiovascular risk factors and disease prevention strategies in primary care among residents of Yinzhou, China. In practice, the Chinese CVD risk-assessment guidelines recommended a specific risk classification method based on the importance of risk factors on 10-year CVD risk, identified in two major cohort studies in China.[21] However, these cohorts were accrued decades ago and may not reflect the contemporary experience of Chinese population. That is, the rapid economic transformation (industrialization, marketization, urbanization, and globalization) in China has contributed to aging populations, unhealthy lifestyles, environmental changes, and epidemiological transitions.[3] In addition, although recently published China-PAR (Prediction for ASCVD Risk in China) model,[22] including traditional CVD risk factors for CVD prediction in Chinese, could be the potential tool, this has not been independently validated and not implemented in real clinical practice. Disparities in risk factor distributions, baseline survival, and composition of disease subtypes were observed within China. Cutoffs to be used for 5- and 10-year risk predictions in Chinese population require more evidence from real-word circumstances. The TRIPOD guidelines also recommended that prediction models using real-world data should be developed.[17] We, therefore, aim to search for the up-to-date CVD risk assessment tool for cardiovascular disease among a Chinese population under the current level of economic development in real-world clinical practice settings. We will also provide evidence for different screening strategies in a real-world circumstance. We expect that these data will be useful in

For peer review only - http://bmjopen.bmj.com/site/about/guidelines.xhtml

supporting a wide range of cardiovascular epidemiology and public health research.

Linking big data from EHRs to a population-based cohort will be a powerful tool for investigating quality of care and improving cardiovascular outcomes. Big data studies in developed countries are generally robust. For example, the CALIBER[4] in the United Kingdom is based on integrated health care databases with both nationwide EHRs in primary care and ongoing national quality registries; Sweden has similar health systems, but its primary care is organized regionally.[23] In addition, regional primary and ambulatory care data are also available for research linkages in the CANHEART study in Ontario, Canada.[5] Unfortunately, large-scale big data research on CVD that is based on EHRs is currently underrepresented in China.[24] Although cardiovascular disease registries in China, such as the Chinese National Stroke Registry (CNSR),[25] have been invaluable to drive research for secondary prevention of CVD, the evidence is often lacking for primary prevention in general populations. China's recent development in big data could facilitate EHR-based epidemiological studies of CVD, especially in some developed regions, such as Xiamen (Fujian Province) and Minhang District (Shanghai). However, little is known to understand the EHR landscape in China because of a lack of published protocols and low levels of data accessibility to these administrative healthcare databases.[26] The CHERRY study has been inspired by all these studies, but differs in terms of its outstanding GP-based primary care units and unique integrated information system. To our knowledge, the CHERRY study is among the first in China to establish a research platform by linking big data across primary and secondary care and disease surveillance. In particular, it is unique in the ability to trace a complete lifetime health care journey in one million adults in a general Chinese population. CHERRY uses coded and inherently linked EHRs from primary care, hospitals, disease surveillance, and ultimately, death registries. Particularly, many EHR-based cohorts in developed countries do not have complete information on lifestyle factors (e.g., smoking status, alcohol use, and others),[27] whereas 87%

For peer review only - http://bmjopen.bmj.com/site/about/guidelines.xhtml

of the population has at least one measurement of smoking status or alcohol use in CHERRY (**Table 3**).

It is known that less than 10 potentially modifiable risk factors might account for more than 90% of the population attributable risk of CVD worldwide.[28 29] However, disparities in the effects of individual risk factors on CVD have also been found across different populations. Although the prevalence of CVD is declining in many developed countries with effective risk-lowering strategies for cardiovascular risk factors, such as smoking cessation or salt reduction, the prevalence of CVD in China is still increasing. The current focus on CVD prevention in the latest guidelines emphasizes the use of risk assessment for appropriate prevention strategies aimed at those with a high risk of CVD.[30] This is consistent with the objectives of the CHERRY study.

According to the checklist of the TRIPOD guidelines,[17] prediction models using real-world data should be developed to discriminate the risk of death and events, and should be used in cost-effectiveness decision models for both public health and clinical practice. The CHERRY cohort will be established as a resource to encourage collaborative research based on individual participant data, so as to strengthen prognostic model development and external validation. For instance, recently published China-PAR models[22] for CVD prediction in Chinese populations could be validated in the CHERRY cohort. Furthermore, large-scale EHR-based cohort studies with detailed longitudinal measurements of clinical risk and patient care data over time are warranted, to better understand how risk factors influence the onset and progression of stroke and other CVDs. There are unique opportunities for the development of dynamic risk prediction models.[31] The CHERRY study can be leveraged to develop risk prediction that is more organic, iterative, and contemporary.

For peer review only - http://bmjopen.bmj.com/site/about/guidelines.xhtml

The study has some limitations. EHR data are known to suffer from a variety of data quality problems. Conflicting data across different sources in EHR-based data also exist in CHERRY. In CALIBER study,[32] the completeness and diagnostic validity of myocardial infarction recording varied across four EHRs sources in primary care, hospital care, disease registry, and mortality register. 31.0% of patients with non-fatal acute myocardial infarction was recorded in three out of four sources and 63.9% in at least two sources. Each data source missed a substantial proportion (25-50%) of myocardial infarction events. A similar situation occurred in CHERRY. In addition, multiple records with similar but slightly different times of diagnosis for one patient may be recorded from different sources owing to varying timing accuracy. Prioritization of sources in terms of conflicting data will be set up. Disease surveillance was considered as gold standard. Events for one patient within a certain time range will be considered a single event; the allowed time window is disease-specific. Secondly, missing data is one of the main limitations of any EHR-converted research platform in terms of data quality. In CHERRY, data completeness varies [e.g., 85.47% of people have at least one record on body mass index (BMI) measurement and 79.07% have their educational level recorded in the system (**Table 3**)]. Developments in imputation within longitudinal cohorts may offer an alternative solution. Finally, although the study has a relatively large number of participants, it is a regional study that is located in a developed area of China. The study population is therefore not nationally representative.

Ethics and dissemination

EHR use is becoming routine. Responsible data sharing is currently being defined, with principles established and policies set globally, such as the Health Insurance Portability and Accountability Act (HIPAA) and the Health Information Technology for Economic and Clinical Health (HITECH) Act in the United States. Security, privacy, confidentiality, and informed-consent issues are being carefully studied by many parties, and solutions are still in progress.[33] As China currently has not set its

For peer review only - http://bmjopen.bmj.com/site/about/guidelines.xhtml

own standards nor defined implementation specifications and certification criteria for EHR use, as in the HIPAA and the HITECH Act, researchers in China can apply to the local health authority (Health and Family Planning Bureau of Yinzhou District) for information on EHR data for health research purposes, as well as seeking approval by institutional review boards (IRBs) based on international standards. For language and security reasons, foreign researchers are encouraged to apply through their Chinese partners, to facilitate international research collaborations. Although participants in the system are not provided with informed-consent as their information is routinely collected health data, the administrative data are inherently linked using unique encrypted identifiers to ensure privacy and confidentiality by the third-party company (Wonders Information Co., Ltd.). The CHERRY study has been approved by the Peking University Institutional Review Board (IRB00001052-16011) and the local health authority. Results of the study will be disseminated through published journal articles, conferences and seminar presentations. More details will be published on the study website (http://www.cherry-study.org).

In summary, the CHERRY study has the potential to provide population-based insights into the quality and outcomes of cardiovascular care. CHERRY will serve to decrease the burden of obtaining data in a research-ready format and encourage research collaboration.

For peer review only

For peer review only - http://bmjopen.bmj.com/site/about/guidelines.xhtml

References

1. GBD 2016 Causes of Death Collaborators. Global, regional, and national age-sex specific mortality for 264 causes of death, 1980-2016: a systematic analysis for the Global Burden of Disease Study 2016. *Lancet* 2017;390(10100):1151-210.

2. Roth GA, Huffman MD, Moran AE, et al. Global and regional patterns in cardiovascular mortality from 1990 to 2013. *Circulation* 2015;132(17):1667-78.

3. Wu Y, Benjamin EJ, MacMahon S. Prevention and Control of Cardiovascular Disease in the Rapidly Changing Economy of China. *Circulation* 2016;133(24):2545-60.

4. Denaxas SC, George J, Herrett E, et al. Data resource profile: cardiovascular disease research using linked bespoke studies and electronic health records (CALIBER). *Int J Epidemiol* 2012;41(6):1625-38.

5. Tu JV, Chu A, Donovan LR, et al. The Cardiovascular Health in Ambulatory Care Research Team (CANHEART): using big data to measure and improve cardiovascular health and healthcare services. *Circ Cardiovasc Qual Outcomes* 2015;8(2):204-12.

6. Vasan RS, Benjamin EJ. The Future of Cardiovascular Epidemiology. *Circulation* 2016;133(25):2626-33.

7. Rumsfeld JS, Joynt KE, Maddox TM. Big data analytics to improve cardiovascular care: promise and challenges. *Nat Rev Cardiol* 2016;13(6):350-9.

8. Benchimol EI, Smeeth L, Guttmann A, et al. The REporting of studies Conducted using Observational Routinely-collected health Data (RECORD) statement. *PLoS Med* 2015;12(10):e1001885.

9. Blumenthal D, Hsiao W. Lessons from the East--China's rapidly evolving health care system. *N Engl J Med* 2015;372(14):1281-5.

10. Babiarz KS, Miller G, Yi H, et al. New evidence on the impact of China's New

For peer review only

For peer review only - http://bmjopen.bmj.com/site/about/guidelines.xhtml

Rural Cooperative Medical Scheme and its implications for rural primary healthcare: multivariate difference-in-difference analysis. *BMJ* 2010;341:c5617.

11. Hicks KA, Tcheng JE, Bozkurt B, et al. 2014 ACC/AHA Key Data Elements and Definitions for Cardiovascular Endpoint Events in Clinical Trials: A Report of the American College of Cardiology/American Heart Association Task Force on Clinical Data Standards (Writing Committee to Develop Cardiovascular Endpoints Data Standards). *Circulation* 2015;132(4):302-61.
12. Wang JB, Gu MJ, Shen P, et al. Body Mass Index and Mortality: A 10-Year Prospective Study in China. *Sci Rep* 2016;6:31609.
13. Solenkova NV, Newman JD, Berger JS, et al. Metal pollutants and cardiovascular disease: mechanisms and consequences of exposure. *Am Heart J* 2014;168(6):812-22.
14. Zhang Y, Ji X, Ku T, et al. Heavy metals bound to fine particulate matter from northern China induce season-dependent health risks: A study based on myocardial toxicity. *Environ Pollut* 2016;216:380-90.
15. Chen R, Wang C, Meng X, et al. Both low and high temperature may increase the risk of stroke mortality. *Neurology* 2013;81(12):1064-70.
16. Taljaard M, Tuna M, Bennett C, et al. Cardiovascular Disease Population Risk Tool (CVDPoRT): predictive algorithm for assessing CVD risk in the community setting. A study protocol. *BMJ Open* 2014;4(10):e006701.
17. Collins GS, Reitsma JB, Altman DG, et al. Transparent reporting of a multivariable prediction model for individual prognosis or diagnosis (TRIPOD): the TRIPOD statement. *BMJ* 2015;350:g7594.
18. Woodward M, Tunstall-Pedoe H, Peters SA. Graphics and statistics for cardiology: clinical prediction rules. *Heart* 2017;103(7):538-45.
19. D'Agostino RB, Sr., Vasan RS, Pencina MJ, et al. General cardiovascular risk profile for use in primary care: the Framingham Heart Study. *Circulation* 2008;117(6):743-53.

For peer review only - http://bmjopen.bmj.com/site/about/guidelines.xhtml

20. Wu N, Tang X, Wu Y, et al. Cohort profile: the Fangshan Cohort Study of cardiovascular epidemiology in Beijing, China. *J Epidemiol* 2014;24(1):84-93.

21. Zhao D, Liu J, Xie W, et al. Cardiovascular risk assessment: a global perspective. *Nat Rev Cardiol* 2015;12(5):301-11.

22. Yang X, Li J, Hu D, et al. Predicting the 10-Year Risks of Atherosclerotic Cardiovascular Disease in Chinese Population: The China-PAR Project (Prediction for ASCVD Risk in China). *Circulation* 2016;134(19):1430-40.

23. Chung SC, Sundstrom J, Gale CP, et al. Comparison of hospital variation in acute myocardial infarction care and outcome between Sweden and United Kingdom: population based cohort study using nationwide clinical registries. *BMJ* 2015;351:h3913.

24. Goldstein BA, Navar AM, Pencina MJ, et al. Opportunities and challenges in developing risk prediction models with electronic health records data: a systematic review. *J Am Med Inform Assoc* 2017;24(1):198-208.

25. Wang Y, Cui L, Ji X, et al. The China National Stroke Registry for patients with acute cerebrovascular events: design, rationale, and baseline patient characteristics. *Int J Stroke* 2011;6(4):355-61.

26. Milea D, Azmi S, Reginald P, et al. A review of accessibility of administrative healthcare databases in the Asia-Pacific region. *J Mark Access Health Policy* 2015;3.

27. Birkhead GS, Klompas M, Shah NR. Uses of electronic health records for public health surveillance to advance public health. *Annu Rev Public Health* 2015;36:345-59.

28. Yusuf S, Hawken S, Ounpuu S, et al. Effect of potentially modifiable risk factors associated with myocardial infarction in 52 countries (the INTERHEART study): case-control study. *Lancet* 2004;364(9438):937-52.

29. O'Donnell MJ, Chin SL, Rangarajan S, et al. Global and regional effects of potentially modifiable risk factors associated with acute stroke in 32

For peer review only - http://bmjopen.bmj.com/site/about/guidelines.xhtml

countries (INTERSTROKE): a case-control study. *Lancet* 2016;388(10046):761-75.

30. Yang Q, Zhong Y, Gillespie C, et al. Assessing potential population impact of statin treatment for primary prevention of atherosclerotic cardiovascular diseases in the USA: population-based modelling study. *BMJ Open* 2017;7(1):e011684.

31. Jutte DP, Roos LL, Brownell MD. Administrative record linkage as a tool for public health research. *Annu Rev Public Health* 2011;32:91-108.

32. Herrett E, Shah AD, Boggon R, et al. Completeness and diagnostic validity of recording acute myocardial infarction events in primary care, hospital care, disease registry, and national mortality records: cohort study. *BMJ* 2013;346:f2350.

33. Kush R, Goldman M. Fostering responsible data sharing through standards. *N Engl J Med* 2014;370(23):2163-5.

For peer review only

For peer review only - http://bmjopen.bmj.com/site/about/guidelines.xhtml

Acknowledgments

The authors thank the Health and Family Planning Bureau of Yinzhou District for providing access to the administrative databases used in the study. We thank Analisa Avila, ELS, from Liwen Bianji, Edanz Group China (www.liwenbianji.cn/ac), for editing the English text of a draft of this manuscript.

Authors' contributions

HL, XT, and PG drafted the manuscript. HL, XT, PS, and PG conceived and designed the study. DZ, JW, JZ, PL, and YS made substantial contributions to the study design. HL, JW, and PS are responsible for study coordination; XT, PS, and JZ are responsible for data quality control; DZ, PL, and YS are responsible for data wrangling; XT, DZ, YS, and PG are responsible for data analysis. All authors contributed to the writing of the study protocol in an iterative manner, and have read and approved the final manuscript.

Funding

This study is supported by the National Natural Science Foundation of China (91546120, 81573226), and the National Thousand Talents Program for Distinguished Young Scholars, China (QNQR201501).

Competing interests

None declared.

For peer review only - http://bmjopen.bmj.com/site/about/guidelines.xhtml

Tables

Table 1 List of core risk factors for cardiovascular disease in the CHERRY study

	Population census and registered health insurance database	Health checks database	Disease surveillance and management database	Outpatient/inpatient EMR database (including laboratory testing)	Charge and claims database	Environmental monitoring database
**Individual-level measurements**						
• Date of entry of first registration	+					
• Date of the measurements		*	*	*	*	
• Date of birth, sex and ethnic groups	+	*		*	*	
• Marital status, education, occupation and socio-economic status (household income, living space, etc.)	+			*		
• Smoking and alcohol use (current/former/never; amount/duration etc.)	+	*	*			
• Physical activity	+	*	*			
• Weight, height, waist and hip circumference	+	*	*			
• History of hypertension and diabetes mellitus	+	*	*	*		
• Prior history of coronary heart disease (in particular myocardial infarction and angina), stroke, transient ischemic attack (TIA), and peripheral vascular disease (PVD)	+	*	*	*		
• Family history of diseases	+		+			
• Systolic and diastolic blood pressure	+	*	*			
• Metabolic factors (including fasting glucose, post-load glucose, and glycosylated hemoglobin)		*	*	*		
• Lipid profiles: total, high- and low-density lipoprotein cholesterol; triglycerides (including information about fasting status at time blood samples were taken);		*	*	*		

For peer review only

For peer review only - http://bmjopen.bmj.com/site/about/guidelines.xhtml

• Blood urea nitrogen, creatinine, uric acid	*	*	*		
• Use of cardiovascular medications (including anti-hypertensive drugs, ‘statins’, fibrates) and other medications (e.g., hypoglycemic agents, hormone replacement therapy)		*	*	*	
• Inflammatory markers (including homocysteine, C-reactive protein, fibrinogen, albumin, interleukin-6 and the leucocyte count)	*		*		
• Haemostatic factors (including von-Willebrand factor and fibrin D-dimer)	*		*		
• Novel CVD related markers (e.g. NT-proBNP)			*		
• Electrocardiogram (ECG)	*		*		
• Cardiovascular imaging information (e.g. progression of coronary artery calcium, carotid intima-media thickness)	*		*		
• Urine Albumin-to-Creatinine Ratio (UACR)	*	*	*		
• Cost of outpatient or inpatient admission (including fees for diagnosis, prescription, laboratory test, surgery etc.)			*	*	
**Environmental and ecological data**					
• Date and region of surveillance					*
• Air temperature and precipitation					*
• Particles with aerodynamic diameter <2.5 µm ($PM_{2.5}$)					*
• Heavy metal concentration in water (lead, cadmium, mercury and arsenic)					*

(+) indicates the database has only one record for the measurements.
(*) indicates the database has multiple records for the measurements.
Note: Health checks database refers to Health checks for New Rural Cooperative Medical Scheme, Health checks for elderly people, and Health checks for adults with hypertension and diabetes in **Figure 2**.

For peer review only

For peer review only - http://bmjopen.bmj.com/site/about/guidelines.xhtml

Table 2 Definitions of major outcomes in the CHERRY study

Events of interest	ICD-10 Code
**Primary events of interest**	
Death due to	
Ischemic heart disease	I20-I25
Cerebrovascular disease	I60-69
Major cardiovascular disease	I00-I78
All-cause mortality	
Hospitalization with main diagnosis of	
Myocardial infarction	I21, I22
Stroke	I60, I61, I63 (excluding I63.6), I64, H34.1
Congestive heart failure	I50
Outpatient visit with main diagnosis of	
Hypertension*	I10, I11, I12, I13, I15
Diabetes mellitus†	E10, E11, E13, E14
Cardiovascular disease	I00-I99
**Secondary events of interest (fatal and non-fatal)**	
ST-Elevation Myocardial Infarction (STEMI)	R94.30
Non-ST segment elevation myocardial infarction (NSTEMI)	R94.31
Ischemic stroke	I63, I64, H34.1 (excluding I63.6)
Hemorrhagic stroke	I60, I61
Transient ischemic attack	G45 (excluding G45.4), H34.0
Atrial fibrillation	I48
Aortic aneurysm / aortic dissection	I71
Peripheral artery disease	I70.2, I73.9, I74.3, I74.4

* Hypertension includes either registered in the hypertension management system, or self-reported history of hypertension during health checks, or hospital admission with a diagnosis of hypertension.

† Diabetes mellitus includes either registered in the diabetes management system, or self-reported history of diabetes during health checks, or hospital admission with a diagnosis of diabetes.

For peer review only

For peer review only - http://bmjopen.bmj.com/site/about/guidelines.xhtml

Table 3 Characteristics of participants in the CHERRY study, by sex

	Men		Women		Overall	
	***N*=512,043**	**% having at least one measurement**	***N*=541,522**	**% having at least one measurement**	***N*=1,053,565**	**% having at least one measurement**
**Age of first registration** (years)	40.17 ± 15.17	100%	39.24 ± 15.56	100%	39.69 ± 15.38	100%
**Region** [n (%)]		98.93%		98.45%		98.68%
Rural	354,524(69.99%)		364,120(68.30%)		718,644(69.12%)	
Urban	152,038(30.01%)		169,017(31.70%)		321,055(30.88%)	
**Education** [n (%)]		79.05%		79.10%		79.07%
Junior high school or lower	324,438(80.16%)		352,534(82.30%)		676,972(81.26%)	
Senior high school or higher	80,307 (19.84%)		75,801(17.70%)		156,108(18.74%)	
**Smoking status** [n (%)]		88.79%		85.60%		87.15%
Never smoker	271,870(59.80%)		440,834(95.11%)		712,704(77.62%)	
Former smoker	23,099(5.08%)		2,828(0.61%)		25,927(2.82%)	
Current smoker	159,664(35.12%)		19,861(4.28%)		179,525(19.55%)	
**Alcohol use** [n (%)]		88.74%		85.57%		87.11%
≥3 days per week	87,064(19.16%)		3,608(0.78%)		90,672(9.88%)	
<3 days per week	43,811(9.64%)		3,713(0.80%)		47,524(5.18%)	
Never drinker	323,518(71.20%)		456,059(98.42%)		779,577(84.94%)	
**Body mass index (BMI)** [kg/m^2]	22.71 ± 2.32	86.97%	22.25 ± 2.57	84.06%	22.47 ± 2.46	85.47%
**Systolic blood pressure** [mmHg]	128.09 ± 14.56	41.37%	126.16 ± 15.11	40.53%	127.11 ± 14.87	40.94%
**Diastolic blood pressure** [mmHg]	79.89 ± 9.76	41.35%	78.13 ± 10.02	40.52%	79.00 ± 9.93	40.93%

For peer review only - http://bmjopen.bmj.com/site/about/guidelines.xhtml

Figure legends

Figure 1 Study location for the CHinese Electronic Health Records Research in Yinzhou (CHERRY) study

Figure 2 Data sources for establishing the CHinese Electronic health Records Research in Yinzhou (CHERRY) cohort

Notes: Although the main focus of the CHERRY study is on adults, data sources of infants, children, and pregnant women are included in the health information system, e.g. birth weight from birth certificates. However, birth records are generally not available for adults who were already over 18 years old in 2009. Maternal exposures from antenatal examination records, i.e. gestational hypertension or diabetes, were recorded in the system which can be potential risk factors for CVD prediction in women. However, a further ethics review process is required to extract maternal information in CHERRY.

For peer review only

For peer review only - http://bmjopen.bmj.com/site/about/guidelines.xhtml

Supplementary files

Figure S1. Administrative databases for the CHERRY cohort

Note: Datasets listed in green in the system are currently not included in the CHERRY cohort.

Table S1. Description of CHERRY study data sources and dataset codes

Table S2. Information on cardiovascular disease diagnosis in different data sources for the CHERRY study

For peer review only

For peer review only - http://bmjopen.bmj.com/site/about/guidelines.xhtml

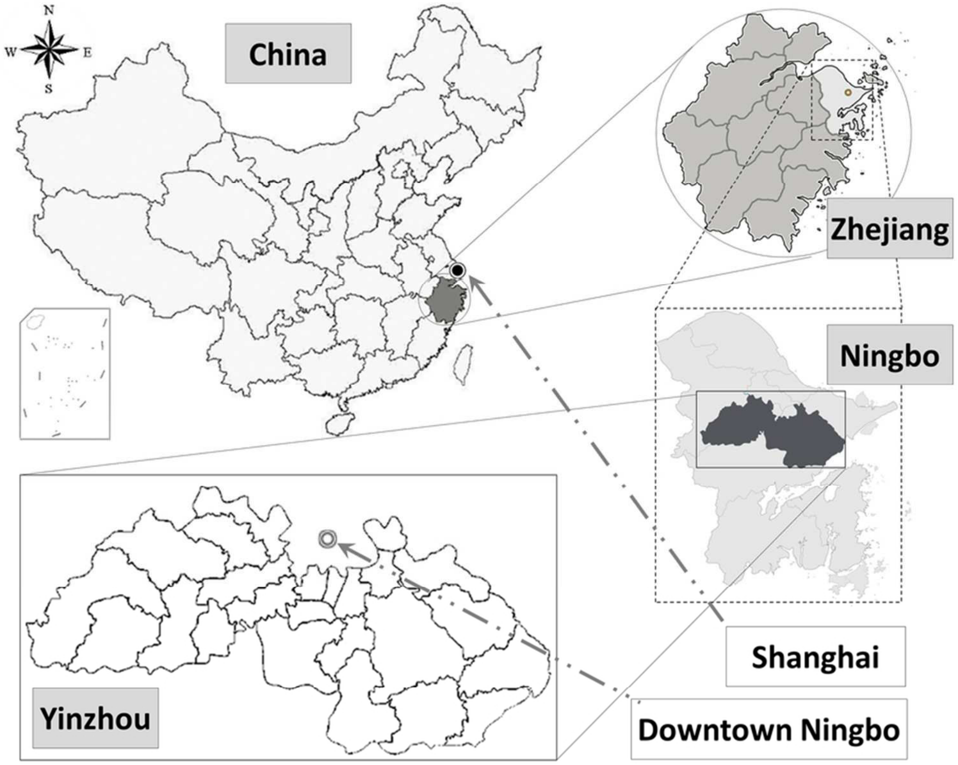

Study location for the CHinese Electronic Health Records Research in Yinzhou (CHERRY)

80x64mm (300 x 300 DPI)

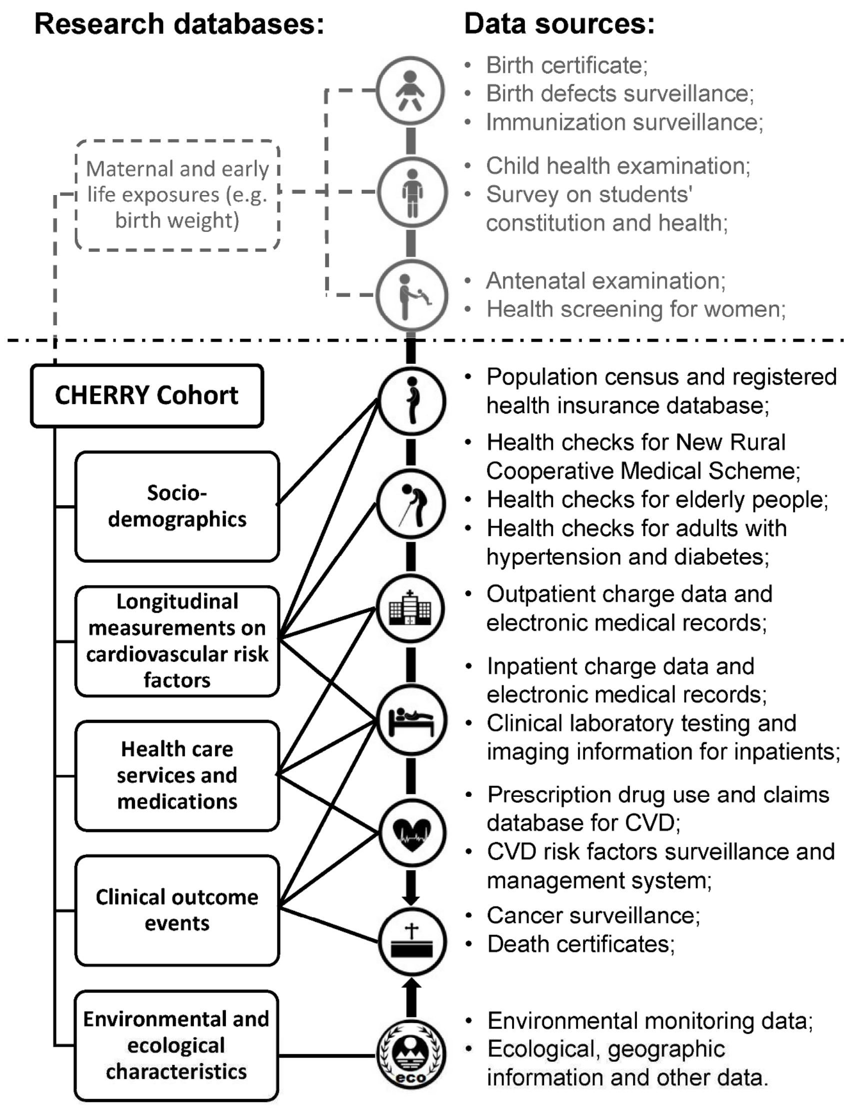

Notes: Although the main focus of the CHERRY study is on adults, data sources of infants, children, and pregnant women are included in the health information system, e.g. birth weight from birth certificates. However, birth records are generally not available for adults who were already over 18 years old in 2009. Maternal exposures from antenatal examination records, i.e. gestational hypertension or diabetes, were recorded in the system which can be potential risk factors for CVD prediction in women. However, a further ethics review process is required to extract maternal information in CHERRY.

210x298mm (300 x 300 DPI)

For peer review only - http://bmjopen.bmj.com/site/about/guidelines.xhtml

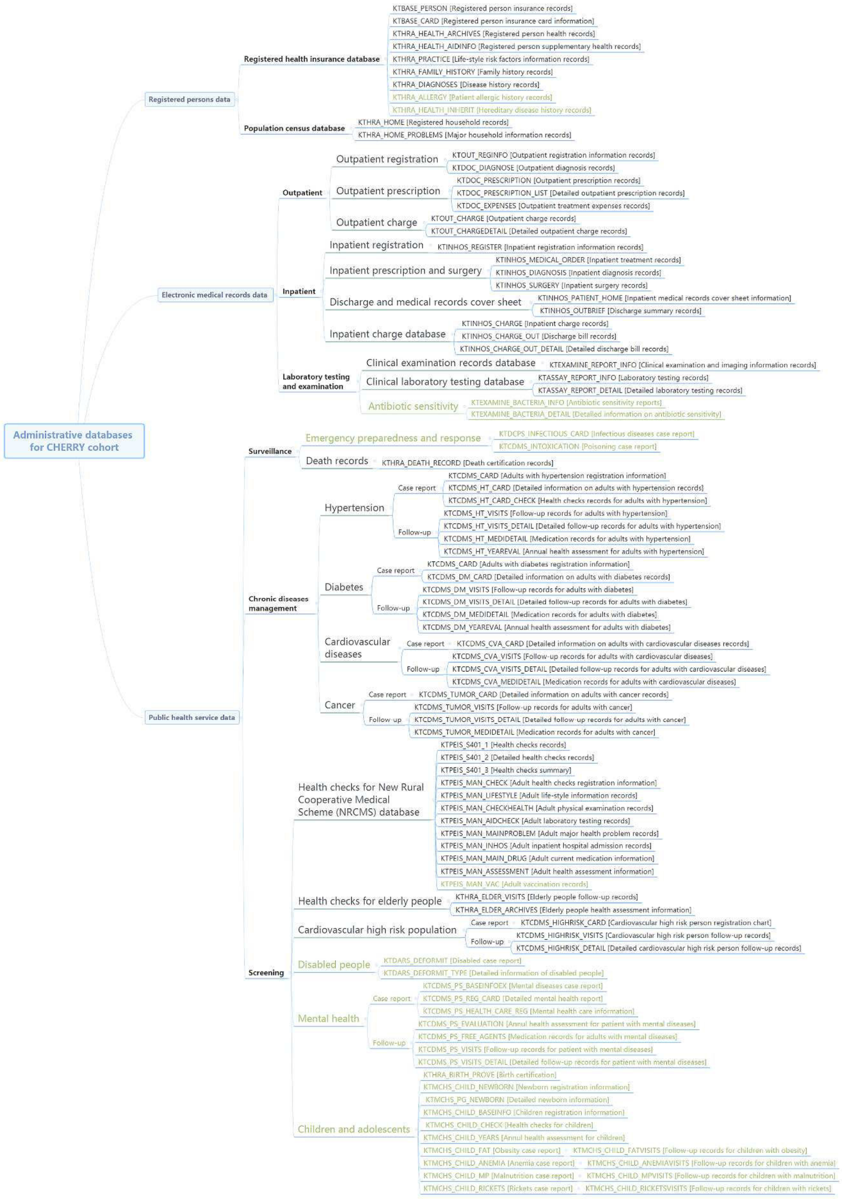

210x297mm (300 x 300 DPI)

Supplemental files

Table S1. Description of CHERRY study data sources and dataset codes

Research databases	Data sources	Dataset codes	Description
Socio-demographics	Registered health insurance database	KTBASE_PERSON	Registered person insurance records
		KTBASE_CARD	Registered person insurance card information
		KTHRA_HEALTH_ARCHIVES	Registered person health records
		KTHRA_HEALTH_AIDINFO	Registered person supplementary health records
		KTHRA_PRACTICE	Life-style risk factors information records
		KTHRA_FAMILY_HISTORY	Family history records
		KTHRA_DIAGNOSES	Disease history records
	Population census database	KTHRA_HOME	Registered household records
		KTHRA_HOME_PROBLEMS	Major household information records

For peer review only - http://bmjopen.bmj.com/site/about/guidelines.xhtml

Longitudinal measurements on cardiovascular risk factors	Health checks for New Rural Cooperative Medical Scheme (NRCMS) database	KTPEIS_S401_1	Health checks records
		KTPEIS_S401_2	Detailed health checks records
		KTPEIS_S401_3	Health checks summary
		KTPEIS_MAN_CHECK	Adult health checks registration information
		KTPEIS_MAN_LIFESTYLE	Adult life-style information records
		KTPEIS_MAN_CHECKHEALTH	Adult physical examination records
		KTPEIS_MAN_AIDCHECK	Adult laboratory testing records
		KTPEIS_MAN_MAINPROBLEM	Adult major health problem records
		KTPEIS_MAN_INHOS	Adult inpatient hospital admission records
		KTPEIS_MAN_MAIN_DRUG	Adult current medication information
		KTPEIS_MAN_ASSESSMENT	Adult health assessment information

For peer review only

	Health checks for elderly people database	KTHRA_ELDER_VISITS	Elderly people follow-up records
		KTHRA_ELDER_ARCHIVES	Elderly people health assessment information
	Health checks for adults with hypertension database	KTCDMS_CARD	Adults with hypertension registration information
		KTCDMS_HT_CARD	Detailed information on adults with hypertension records
		KTCDMS_HT_CARD_CHECK	Health checks records for adults with hypertension
		KTCDMS_HT_VISITS	Follow-up records for adults with hypertension
		KTCDMS_HT_VISITS_DETAIL	Detailed follow-up records for adults with hypertension
		KTCDMS_HT_MEDIDETAIL	Medication records for adults with hypertension
		KTCDMS_HT_YEAREVAL	Annual health assessment for adults with hypertension
	Health checks for adults with diabetes database	KTCDMS_CARD	Adults with diabetes registration information
		KTCDMS_DM_CARD	Detailed information on adults with diabetes records

For peer review only - http://bmjopen.bmj.com/site/about/guidelines.xhtml

	KTCDMS_DM_VISITS	Follow-up records for adults with diabetes
	KTCDMS_DM_VISITS_DETAIL	Detailed follow-up records for adults with diabetes
	KTCDMS_DM_MEDIDETAIL	Medication records for adults with diabetes
	KTCDMS_DM_YEAREVAL	Annual health assessment for adults with diabetes
Cardiovascular high-risk population	KTCDMS_HIGHRISK_CARD	Cardiovascular high-risk person registration chart
	KTCDMS_HIGHRISK_VISITS	Cardiovascular high-risk person follow-up records
	KTCDMS_HIGHRISK_DETAIL	Detailed cardiovascular high-risk person follow-up records
Clinical examination records database	KTEXAMINE_REPORT_INFO	Clinical examination and imaging information records
Clinical laboratory testing database	KTASSAY_REPORT_INFO	Laboratory testing records
	KTASSAY_REPORT_DETAIL	Detailed laboratory testing records

For peer review only

For peer review only - http://bmjopen.bmj.com/site/about/guidelines.xhtml

Health care services and medications	Outpatient medical records database	KTOUT_REGINFO	Outpatient registration information records
		KTDOC_DIAGNOSE	Outpatient diagnosis records
	Outpatient prescription database	KTDOC_PRESCRIPTION	Outpatient prescription records
		KTDOC_PRESCRIPTION_LIST	Detailed outpatient prescription records
		KTDOC_EXPENSES	Outpatient treatment expenses records
	Outpatient charge database	KTOUT_CHARGE	Outpatient charge records
		KTOUT_CHARGEDETAIL	Detailed outpatient charge records
	Inpatient medical records database	KTINHOS_REGISTER	Inpatient registration information records
		KTINHOS_PATIENT_HOME	Inpatient medical records cover sheet information
		KTINHOS_OUTBRIEF	Discharge summary records

For peer review only

	Inpatient prescription database	KTINHOS_MEDICAL_ORDER	Inpatient treatment records
		KTINHOS_DIAGNOSIS	Inpatient diagnosis records
		KTINHOS_SURGERY	Inpatient surgery records
	Inpatient charge database	KTINHOS_CHARGE	Inpatient charge records
		KTINHOS_CHARGE_OUT	Discharge bill records
		KTINHOS_CHARGE_OUT_DETAIL	Detailed discharge bill records
Clinical outcome events	Cardiovascular diseases surveillance and management system	KTCDMS_CVA_CARD	Detailed information on adults with cardiovascular disease records
		KTCDMS_CVA_VISITS	Follow-up records for adults with cardiovascular diseases
		KTCDMS_CVA_VISITS_DETAIL	Detailed follow-up records for adults with cardiovascular diseases
		KTCDMS_CVA_MEDIDETAIL	Medication records for adults with cardiovascular diseases

For peer review only - http://bmjopen.bmj.com/site/about/guidelines.xhtml

Cancer surveillance database	KTCDMS_TUMOR_CARD	Detailed information on adults with cancer records
	KTCDMS_TUMOR_VISITS	Follow-up records for adults with cancer
	KTCDMS_TUMOR_VISITS_DETAIL	Detailed follow-up records for adults with cancer
	KTCDMS_TUMOR_MEDIDETAIL	Medication records for adults with cancer
Database of death records	KTHRA_DEATH_RECORD	Death certificates

For peer review only

Table S2. Information on cardiovascular disease diagnosis in different data sources for the CHERRY study

	Clinical diagnosis	ICD-10 Code	Clinical symptom		Neurological examination	Cerebrospinal fluid (CSF) analysis	Serum enzyme tests	Electrocardiogram (ECG)	Electroencephalogram (EEG)	Coronary computed tomography angiography (CTA)	Computed tomography (CT) / magnetic resonance imaging (MRI)		Autopsy
			Brief	Detail							Summary	Imaging	
Disease surveillance / Death certificates (gold standard)	√	√	√				√	√	√	√	√		√
Disease management database (primary care)	√	√	√	√			√	√	√		√		
Electronic medical records database (hospital care)	√	√	√	√	√	√	√	√	√	√	√	√	
Health insurance database	√	√											

For peer review only

BMJ Open

Using big data to improve cardiovascular care and outcomes in China: a protocol for the CHinese Electronic health Records Research in Yinzhou (CHERRY) study

Journal:	*BMJ Open*
Manuscript ID	bmjopen-2017-019698.R2
Article Type:	Protocol
Date Submitted by the Author:	21-Dec-2017
Complete List of Authors:	Lin, Hongbo; Yinzhou District Center for Disease Control and Prevention<br>TANG, Xun; Peking University Health Science Center, Department of Epidemiology and Biostatistics<br>Shen, Peng; Yinzhou District Center for Disease Control and Prevention<br>Zhang, Dudan; Peking University Health Science Center, Department of Epidemiology and Biostatistics<br>Wu, Jinguo; Wonders Information Co., Ltd.<br>Zhang, Jingyi; Wonders Information Co., Ltd.<br>Lu, Ping; Wonders Information Co., Ltd.<br>Si, Yaqin; Peking University Health Science Center, Department of Epidemiology and Biostatistics<br>GAO, Pei; Peking University Health Science Center, Department of Epidemiology and Biostatistics
**Primary Subject Heading**:	Cardiovascular medicine
Secondary Subject Heading:	Epidemiology
Keywords:	cardiovascular diseases, electronic health records, Chinese

SCHOLARONE™
Manuscripts

Using big data to improve cardiovascular care and outcomes in China: a protocol for the CHinese Electronic health Records Research in Yinzhou (CHERRY) study

Hongbo Lin, [1]* Xun Tang, [2]* Peng Shen, [1] Dudan Zhang, [2] Jinguo Wu, [3] Jingyi Zhang, [3] Ping Lu, [3] Yaqin Si, [2] Pei Gao, [2#]

[1] Yinzhou District Center for Disease Control and Prevention, Ningbo 315100, China

[2] Department of Epidemiology and Biostatistics, Peking University Health Science Center, Beijing 100191, China

[3] Wonders Information Co., Ltd., Shanghai 201112, China

* Drs. Hongbo Lin and Xun Tang contributed equally to this paper.

[#] **Correspondence to:**

Prof. Pei Gao, PhD

Department of Epidemiology and Biostatistics,

Peking University Health Science Center,

38 Xueyuan Road, Beijing 100191, China

Tel/Fax: +86-10-82805642

Email: peigao@bjmu.edu.cn

Total word count: 5020 words, Abstract: 298 words, 2 Figures and 3 Tables, 34 References, 3 Supplementary files

For peer review only - http://bmjopen.bmj.com/site/about/guidelines.xhtml

Abstract

Introduction: Data based on electronic health records (EHRs) is rich with individual-level longitudinal measurement information and are becoming an increasingly common data source for clinical risk prediction worldwide. However, few EHR-based cohort studies are available in China. Harnessing EHRs for research requires a full understanding of data linkages, management, and data quality in large data sets, which presents unique analytic opportunities and challenges. The purpose of this study is to provide a framework to establish a uniquely integrated EHR database in China for scientific research.

Methods and Analysis: The CHinese Electronic health Records Research in Yinzhou (CHERRY) study will extract individual participant data within the regional health information system of an eastern coastal area of China to establish a longitudinal population-based ambispective cohort study for cardiovascular care and outcomes research. A total 1,053,565 Chinese adults aged over 18 years were registered in the health information system in 2009, and there were 23,394 deaths from 1 January 2009 to 31 December 2015. The study will include information from multiple epidemiological surveys; EHRs for chronic disease management; and health administrative, clinical, laboratory, drug, and electronic medical record databases. Follow-up of fatal and non-fatal clinical events is achieved through records linkage to the regional system of disease surveillance, chronic disease management and electronic medical records (based on diagnostic codes from the International Classification of Diseases [the Tenth Revision]). The CHERRY study will provide a unique platform and serve as a valuable big data resource for cardiovascular risk prediction and population management, for primary and secondary prevention of cardiovascular events in China.

Ethics and dissemination: The CHERRY study was approved by the Peking

For peer review only

University Institutional Review Board (IRB00001052-16011) in April 2016. Results of the study will be disseminated through published journal articles, conferences and seminar presentations, and on the study website (http://www.cherry-study.org).

For peer review only

Strengths and limitations of this study

- The Chinese Electronic health Records Research in Yinzhou (CHERRY) study is a large, natural population-based, observational cohort study linking big data of integrated individual-level electronic health records (EHRs).
- The CHERRY study is among the first in China to establish a research platform from EHRs system for investigating a wide range of important issues regarding primary and secondary prevention of cardiovascular disease in a real-world circumstance.
- The CHERRY study is unique in its ability to trace the complete lifetime health care journey using birth certificates, health checks, primary care visits, hospitalizations, disease surveillance, and ultimately, death certificates for one million adults in the general Chinese population.
- Missing data and conflicting data might be the main limitations of any EHR-converted big data research in terms of data quality; however, imputation for missing data within longitudinal cohorts and setup of the priority of data sources for conflicting data may offer alternative solutions.
- Although the CHERRY study has a relatively large number of participants, it is a regional cohort located in a developed area of China and as such, will not be nationally representative.

For peer review only

For peer review only - http://bmjopen.bmj.com/site/about/guidelines.xhtml

Introduction

Cardiovascular disease (CVD) is the leading global disease burden worldwide.[1] Epidemiological studies have suggested that age-standardized CVD mortality rates have been falling in developed countries in recent decades.[2] However, China's current cardiovascular epidemic is increasing with rapid economic development and changing lifestyles.[3] Some established studies, such as the Cardiovascular disease research using Linked Bespoke studies and Electronic health Records (CALIBER)[4] in the United Kingdom and Cardiovascular Health in Ambulatory Care Research Team (CANHEART)[5] in Canada, have showed that forging a partnership between electronic health records (EHRs) and population-based cohort studies for CVD epidemiology is useful and growing. EHR-based data is rich with individual-level longitudinal measurement information. However, few existing studies in China to date have successfully assembled cohort study based on EHRs at the population level. Success in linking big data to population-based cohort could promote advances both in improving cardiovascular outcomes and facilitating health care services research.[6]

Several critical factors affect the quality of EHR-based data for research, e.g., appropriate approaches to data management and linkages, operational definitions for exposures, ascertainment and adjudication of outcomes, methods for handling missing data, and valid interpretation of clinical or public health integration and utility. These are all essential to generating robust findings.[7,8] Consequently, in this protocol, we aimed to describe the detailed methods and data sources used to establish an EHR-based general population-based cohort study in an eastern coastal area of China, the CHinese Electronic health Records Research in Yinzhou (CHERRY) study. By linking various data sets at the individual level using unique and encoded identifiers, the CHERRY study will be used as a research platform to address several important research questions, including but not limited to the following objectives:

For peer review only - http://bmjopen.bmj.com/site/about/guidelines.xhtml

1. What are the emerging risk factors, assembling both individual-level and community-level characteristics, for the incidence of major cardiovascular events in this developed area of China?
2. What is the up-to-date and suitable CVD risk assessment model to use for Chinese population in nutrition transition?
3. How do the different screening strategies for targeting people at high risk of CVD perform in a real-world circumstance?
4. How does the regional health system deliver various CVD prevention practices in terms of cardiovascular outcome improvement?

Methods and analysis

The CHERRY study is a unique, population-based observational research resource that is aimed at improving cardiovascular care and outcomes in China. A particular focus will be on cardiovascular risk prediction and population management, providing evidence to improve the primary and secondary prevention of cardiovascular events.

Study area

Yinzhou is a district of Ningbo in Zhejiang province (29°37′-29°57′ N, 121°08′-121°54′ E), located 230 kilometers south of Shanghai, China, with an area of 1,346 km^2 (**Figure 1**). Yinzhou had a total population of 1.24 million in 2016, including 790,600 permanent residents. This particular region is a developed area and was selected because of its outstanding general practice based primary care units and unique integrated electronic health information system. Particularly, the system has captured rich information from primary care units, including important information on cardiovascular risk factors and their disease management among communities. This enables us to use comprehensive big data for both

For peer review only - http://bmjopen.bmj.com/site/about/guidelines.xhtml

epidemiological and clinical research in China.

Data sources and cohort establishment

Health information systems in Yinzhou include different administrative databases of general demographic characteristics, health check information, inpatient and outpatient electronic medical records (EMRs), health insurance database, disease management, and death certificates, etc. The special feature of the system in Yinzhou is that these databases are inherently linked to each other by a unique and encoded identifier for each individual. The system was originally designed in 2006 by the Yinzhou District Center for Disease Control and Prevention to facilitate routine primary care services for local general practitioners (GPs). It was then gradually integrated with information on public health surveillance, population screening, disease management, health information system in hospitals and other healthcare services. Since 2009, this regional system has covered nearly all health-related activities of residents within this region, from birth to death, including children, adolescents, pregnant women, adults and elderly people (**Figure 2** and **Figure S1**). Now 98% of permanent residents in Yinzhou have registered in the health information system with a valid healthcare identifier.

Consequently, based on the data sources in this integrated system, the CHERRY study started in 2016 and is now extracting individual participants' data within the system to establish a natural, longitudinal, population-based, ambispective cohort study for cardiovascular care and outcomes research. The study comprises all participants registered in the system if they: (1) were over 18 years of age on 1 January 2009; (2) have complete information on date of birth, sex, and a valid healthcare identifier; (3) have been living in Yinzhou for at least 6 months; and (4) are Chinese nationality. We chose 1 January 2009 as the date of cohort inception to bypass the integration and preliminary test period of the system and to allow for full coverage of the regional population. Once included in the CHERRY cohort,

For peer review only - http://bmjopen.bmj.com/site/about/guidelines.xhtml

individuals remain in it until death or termination of local health insurance (primarily owing to moving out of the province). Follow-up is generally continuous in the health information system. CHERRY will update certain important information such as vital status, clinical outcomes, and claims data for all cohort members annually from the administrative databases. A third party, Wonders Information Co., Ltd., was engaged to handle linkage and safe storage of the linked datasets, ensuring privacy protection in the CHERRY study. A description of the CHERRY research cohort in relation to data sources in the administrative health information system is shown in **Figure 2** and **Table S1**. Each source captures a different aspect of a person's lifetime health care journey, as follows.

Socio-demographics

Basic demographic and socioeconomic information of residents stems from the population census and registered health insurance database in the health information system. Key data variables will include date of birth, sex, ethnic group (e.g., Han, Muslim), marital status, education, occupation, and household information such as income, living space, etc.

Longitudinal measurement of cardiovascular risk factors

Local GPs in Yinzhou have built up an impressive scheme on frequent health checks among adults and regular epidemiological surveys as part of primary care routine services over the 10 years after China's healthcare reform was initially launched.[9] According to the New Rural Cooperative Medical Scheme (NRCMS) in China,[10] general health checks for adults in rural areas are conducted once every 2 years by local GPs. CHERRY then includes longitudinal measurements of risk factors related to CVD at the individual level, e.g., smoking status, alcohol use, BMI and other obesity risk factors, and daily physical activity. Furthermore, screening of hypertension and diabetes mellitus in the general population aged 40 years and older is recommended by Chinese clinical practice guidelines and has been

For peer review only - http://bmjopen.bmj.com/site/about/guidelines.xhtml

implemented in Yinzhou since 2009. Especially, lipid levels are frequently measured for the general population within the health check package paid for by employers, as part of employee welfare. Moreover, for adults with a history of hypertension or diabetes or individuals aged 60 years or over, a comprehensive medical examination is scheduled routinely at least once every year, for disease management. In total, 53% of individuals aged 40 years or over had at least one general health check information (e.g., blood pressure measurements) within the system. Detailed information related to CVD risk factors within the examination is extracted to CHERRY, which includes blood measurements on glucose, Hemoglobin A1c (HbA1c), and lipid profiles (total cholesterol, HDL cholesterol, LDL cholesterol, triglycerides) as well as urine testing, and so on. Additional information from outpatient and inpatient EMRs will also be supplemented. Laboratory measurements in EMRs of circulating inflammation markers (e.g., homocysteine, C-reactive protein, albumin, and leucocyte count), novel CVD related markers [e.g., N-terminal pro b-type natriuretic peptide (NT-proBNP)], or cardiovascular imaging information (e.g., progression of coronary artery calcium) are included when available. The core variables for CVD-related factors and longitudinal measurements in the CHERRY study are listed in **Table 1**.

Health care services and medications

Outpatient and inpatient EMRs containing information of patients' health care services and medications will be transferred to CHERRY. EMR data contains physician visits, primary and secondary disease diagnosis, laboratory services, medications (indication, strength and dosage instructions) and so on from a network of 5 hospitals and over 289 primary care units across Yinzhou. The system (and CHERRY study) contained EMRs in all the hospitals (both public and private hospitals) and primary care units within Yinzhou but no pharmacy stores. Therefore, the dispensation of medicine from both hospitals and primary care units are available (prescription medicines are always taken directly from the pharmacy

For peer review only

For peer review only - http://bmjopen.bmj.com/site/about/guidelines.xhtml

within the hospitals/primary care units in China). Both individuals with and without health insurance can access the primary care and hospital services and therefore are all included in the system/study. By the end of 2015, 95.9% (1,010,658/1,053,565) adult participants with the unique identifier had EMRs in the system, receiving at least one clinical service (hospital or primary care). For patients receiving care outside Yinzhou (e.g., patients might go to famous hospitals in Shanghai for certain complex surgical procedures), major non-fatal events occurred (e.g., CVD and cancer, etc) are tracked from both disease surveillance and chronic disease management system. In this case, patients generally reported to local hospital/GPs for after-surgery health check services and drug prescription. Fatal events are tracked from death registry where death certificates issued from hospitals outside Yinzhou are available. For the information related to the primary disease diagnosis and medication used in hospitals outside the region, limited information is also extracted from the health insurance database. In Yinzhou, 95.7% of permanent residents are covered by the national health insurance. Residents of all ages were covered by the system. However, only adults above 18 years of age on 1 January 2009 are included in the CHERRY study.

Clinical outcome events

For each individual, data will be sought on each of the following outcomes and their dates of occurrence: non-fatal myocardial infarction, non-fatal coronary heart disease (CHD), non-fatal stroke, cause-specific mortality (or at least fatal CHD and fatal stroke), and other cardiovascular outcomes. Precise details of the diagnostic criteria used to define cases have been sought from the 2014 American College of Cardiology/American Heart Association Key Data Elements and Definitions for Cardiovascular Endpoint Events in Clinical Trials.[11] Principal analyses will be based on events classified according to the International Classification of Diseases, Tenth Revision (ICD-10) (**Table 2**). In the CHERRY study, for fatal outcomes, attribution of death refers to the primary cause provided by cause-specific mortality on death

For peer review only - http://bmjopen.bmj.com/site/about/guidelines.xhtml

certificates in the health information system. Data undergo annual quality assessments. A description of the death certificates has been reported previously.[12] For non-fatal outcomes, multiple sources exist in the system for the outcome definition, i.e., disease management database (primary care), EMRs database (hospital care), health insurance database, and disease surveillance database (disease registry). We define the disease surveillance database as gold standard. In Yinzhou, CVD, hypertension, diabetes, or cancer cases were required to be reported for disease surveillance and management by local GPs once the diagnoses were confirmed. Diagnosis of these diseases made by the physicians in all regional hospitals will be automatically sent to the local GPs of patients in the system. Criteria used for the diagnosis of incident cardiovascular morbidity in each source were described in **Table S2**. We define a "definite" event if two or more sources excluding health insurance database reported as a case. A "probable" event is defined if any source (including health insurance database) reported as a case. Cross-validation will be further investigated to improve the data quality and diagnostic validity. Primary and secondary prevention patients are defined as those without (primary) or with (secondary) a known history of CVD (**Table 2**).

Environmental and ecological characteristics

For environmental exposure monitoring data, we will include exposures to major water and air pollutants from eight environmental monitoring sites in Yinzhou, including heavy metal contamination and particles with aerodynamic diameter <2.5 μm ($PM_{2.5}$). These have been associated with increased cardiovascular mortality.[13] [14] In addition, various meteorological conditions, such as air temperature and precipitation, from all weather stations across Yinzhou during the study period will also be available. Previous studies in China have demonstrated that both short-term (days) and longer-term (months or years) variations in temperature increase CVD morbidity and mortality.[15]

For peer review only - http://bmjopen.bmj.com/site/about/guidelines.xhtml

Sample size

A total of 1,053,565 Chinese adults aged over 18 years were registered in the health information system. According to sample size requirements for prediction models,[16] for time to event outcomes, the number of participants experiencing the event must exceed 10 times the number of degrees of freedom, where the number of degrees of freedom includes the number of predictors screened for association with the outcome, all dummy variables, non-linear terms and interactions. By the end of 2015, there were 23,394 deaths (12,669 men and 10,725 women) and 14,217 cardiovascular events (7,825 men and 6,392 women) from 1 January 2009 to 31 December 2015. Thus, the sample size is generally sufficient for the CHERRY study.

Data analysis plan

A detailed data analysis plan will follow the checklist in the Transparent reporting of a multivariable prediction model for individual prognosis or diagnosis (TRIPOD) guidelines[17] and recommendations by other studies.[16 18] In brief, data cleaning will proceed before examining exposure-outcome associations. Descriptive statistics will be used to determine values outside a plausible range, then outliers will be set to missing. Multiple imputation will be used to impute missing values for the predictors, where appropriate. CVD prediction models will be developed from sex-specific Cox proportional hazards models. The main CVD risk factors in established prediction models, such as Framingham risk scores,[19] will be retained in our model directly. We will then evaluate whether the predictive capability of the model will be improved by inclusion of additional predictors, using measures of discrimination and reclassification. The clinical performance of the models will be assessed by discrimination *C* statistics, calibration chi-square and plots, net reclassification improvement (NRI) and the integrated discrimination improvement (IDI) index. Cross-validation will be used to evaluate internal consistency; when available, the prediction models will also be evaluated for external validation in

For peer review only - http://bmjopen.bmj.com/site/about/guidelines.xhtml

other independent cohort studies, such as the Fangshan Cohort Study.[20] All statistical analysis will be conducted using the SAS system, version 9.4 (SAS Institute, Cary, NC, USA) and STATA software, version 14.1 (StataCorp LLC, College Station, TX, USA).

Discussion

The CHERRY study is established to use longitudinal measurements of cardiovascular risk factors and disease prevention strategies in primary care among residents of Yinzhou, China. In practice, the Chinese CVD risk-assessment guidelines recommended a specific risk classification method based on the importance of risk factors on 10-year CVD risk, identified in two major cohort studies in China.[21] However, these cohorts were accrued decades ago and may not reflect the contemporary experience of Chinese population. That is, the rapid economic transformation (industrialization, marketization, urbanization, and globalization) in China has contributed to aging populations, unhealthy lifestyles, environmental changes, and epidemiological transitions.[3] In addition, although recently published China-PAR (Prediction for ASCVD Risk in China) model,[22] including traditional CVD risk factors for CVD prediction in Chinese, could be the potential tool, this has not been independently validated and not implemented in real clinical practice. Disparities in risk factor distributions, baseline survival, and composition of disease subtypes were observed within China. Cutoffs to be used for 5- and 10-year risk predictions in Chinese population require more evidence from real-word circumstances. The TRIPOD guidelines also recommended that prediction models using real-world data should be developed.[17] We, therefore, aim to search for the up-to-date CVD risk assessment tool for cardiovascular disease among a Chinese population under the current level of economic development in real-world clinical practice settings. We will also provide evidence for different screening strategies in a real-world circumstance. We expect that these data will be useful in

For peer review only - http://bmjopen.bmj.com/site/about/guidelines.xhtml

supporting a wide range of cardiovascular epidemiology and public health research.

Linking big data from EHRs to a population-based cohort will be a powerful tool for investigating quality of care and improving cardiovascular outcomes. Big data studies in developed countries are generally robust. For example, the CALIBER[4] in the United Kingdom is based on integrated health care databases with both nationwide EHRs in primary care and ongoing national quality registries; Sweden has similar health systems, but its primary care is organized regionally.[23] In addition, regional primary and ambulatory care data are also available for research linkages in the CANHEART study in Ontario, Canada.[5] Unfortunately, large-scale big data research on CVD that is based on EHRs is currently underrepresented in China.[24] Although cardiovascular disease registries in China, such as the Chinese National Stroke Registry (CNSR),[25] have been invaluable to drive research for secondary prevention of CVD, the evidence is often lacking for primary prevention in general populations. China's recent development in big data could facilitate EHR-based epidemiological studies of CVD, especially in some developed regions, such as Xiamen (Fujian Province) and Minhang District (Shanghai). However, little is known to understand the EHR landscape in China because of a lack of published protocols and low levels of data accessibility to these administrative healthcare databases.[26] The CHERRY study has been inspired by all these studies, but differs in terms of its outstanding GP-based primary care units and unique integrated information system. To our knowledge, the CHERRY study is among the first in China to establish a research platform by linking big data across primary and secondary care and disease surveillance. In particular, it is unique in the ability to trace a complete lifetime health care journey in one million adults in a general Chinese population. CHERRY uses coded and inherently linked EHRs from primary care, hospitals, disease surveillance, and ultimately, death registries. Particularly, many EHR-based cohorts in developed countries do not have complete information on lifestyle factors (e.g., smoking status, alcohol use, and others),[27] whereas 87%

For peer review only - http://bmjopen.bmj.com/site/about/guidelines.xhtml

of the population has at least one measurement of smoking status or alcohol use in CHERRY (**Table 3**).

It is known that less than 10 potentially modifiable risk factors might account for more than 90% of the population attributable risk of CVD worldwide.[28 29] However, disparities in the effects of individual risk factors on CVD have also been found across different populations. Although the prevalence of CVD is declining in many developed countries with effective risk-lowering strategies for cardiovascular risk factors, such as smoking cessation or salt reduction, the prevalence of CVD in China is still increasing. The current focus on CVD prevention in the latest guidelines emphasizes the use of risk assessment for appropriate prevention strategies aimed at those with a high risk of CVD.[30] This is consistent with the objectives of the CHERRY study.

According to the checklist of the TRIPOD guidelines,[17] prediction models using real-world data should be developed to discriminate the risk of death and events, and should be used in cost-effectiveness decision models for both public health and clinical practice. The CHERRY cohort will be established as a resource to encourage collaborative research based on individual participant data, so as to strengthen prognostic model development and external validation. For instance, recently published China-PAR models[22] for CVD prediction in Chinese populations could be validated in the CHERRY cohort. Furthermore, large-scale EHR-based cohort studies with detailed longitudinal measurements of clinical risk and patient care data over time are warranted, to better understand how risk factors influence the onset and progression of stroke and other CVDs. There are unique opportunities for the development of dynamic risk prediction models.[31] The CHERRY study can be leveraged to develop risk prediction that is more organic, iterative, and contemporary.

For peer review only - http://bmjopen.bmj.com/site/about/guidelines.xhtml

The study has some limitations. EHR data are known to suffer from a variety of data quality problems. Conflicting data across different sources in EHR-based data also exist in CHERRY. In CALIBER study,[32] the completeness and diagnostic validity of myocardial infarction recording varied across four EHRs sources in primary care, hospital care, disease registry, and mortality register. 31.0% of patients with non-fatal acute myocardial infarction was recorded in three out of four sources and 63.9% in at least two sources. Each data source missed a substantial proportion (25-50%) of myocardial infarction events. A similar situation occurred in CHERRY. In addition, multiple records with similar but slightly different times of diagnosis for one patient may be recorded from different sources owing to varying timing accuracy. Prioritization of sources in terms of conflicting data will be set up. Disease surveillance was considered as gold standard. Events for one patient within a certain time range will be considered a single event; the allowed time window is disease-specific. Secondly, missing data is one of the main limitations of any EHR-converted research platform in terms of data quality. In CHERRY, data completeness varies [e.g., 85.47% of people have at least one record on body mass index (BMI) measurement and 79.07% have their educational level recorded in the system (**Table 3**)]. Developments in imputation within longitudinal cohorts may offer an alternative solution. Finally, although the study has a relatively large number of participants, it is a regional study that is located in a developed area of China. The study population is therefore not nationally representative.

Ethics and dissemination

EHR use is becoming routine. Responsible data sharing is currently being defined, with principles established and policies set globally, such as the Health Insurance Portability and Accountability Act (HIPAA) and the Health Information Technology for Economic and Clinical Health (HITECH) Act in the United States. Security, privacy, confidentiality, and informed-consent issues are being carefully studied by many parties, and solutions are still in progress.[33] As China currently has not set its

For peer review only - http://bmjopen.bmj.com/site/about/guidelines.xhtml

own standards nor defined implementation specifications and certification criteria for EHR use, as in the HIPAA and the HITECH Act, researchers in China can apply to the local health authority (Health and Family Planning Bureau of Yinzhou District) for information on EHR data for health research purposes, as well as seeking approval by institutional review boards (IRBs) based on international standards. For language and security reasons, foreign researchers are encouraged to apply through their Chinese partners, to facilitate international research collaborations. Although participants in the system are not provided with informed-consent as their information is routinely collected health data, the administrative data are inherently linked using unique encrypted identifiers to ensure privacy and confidentiality by the third-party company (Wonders Information Co., Ltd.). The CHERRY study has been approved by the Peking University Institutional Review Board (IRB00001052-16011) and the local health authority. Results of the study will be disseminated through published journal articles, conferences and seminar presentations. More details will be published on the study website (http://www.cherry-study.org).

In summary, the CHERRY study has the potential to provide population-based insights into the quality and outcomes of cardiovascular care. CHERRY will serve to decrease the burden of obtaining data in a research-ready format and encourage research collaboration.

For peer review only

For peer review only - http://bmjopen.bmj.com/site/about/guidelines.xhtml

References

1. GBD 2016 Causes of Death Collaborators. Global, regional, and national age-sex specific mortality for 264 causes of death, 1980-2016: a systematic analysis for the Global Burden of Disease Study 2016. *Lancet* 2017;390(10100):1151-210.

2. Roth GA, Huffman MD, Moran AE, et al. Global and regional patterns in cardiovascular mortality from 1990 to 2013. *Circulation* 2015;132(17):1667-78.

3. Wu Y, Benjamin EJ, MacMahon S. Prevention and Control of Cardiovascular Disease in the Rapidly Changing Economy of China. *Circulation* 2016;133(24):2545-60.

4. Denaxas SC, George J, Herrett E, et al. Data resource profile: cardiovascular disease research using linked bespoke studies and electronic health records (CALIBER). *Int J Epidemiol* 2012;41(6):1625-38.

5. Tu JV, Chu A, Donovan LR, et al. The Cardiovascular Health in Ambulatory Care Research Team (CANHEART): using big data to measure and improve cardiovascular health and healthcare services. *Circ Cardiovasc Qual Outcomes* 2015;8(2):204-12.

6. Vasan RS, Benjamin EJ. The Future of Cardiovascular Epidemiology. *Circulation* 2016;133(25):2626-33.

7. Rumsfeld JS, Joynt KE, Maddox TM. Big data analytics to improve cardiovascular care: promise and challenges. *Nat Rev Cardiol* 2016;13(6):350-9.

8. Benchimol EI, Smeeth L, Guttmann A, et al. The REporting of studies Conducted using Observational Routinely-collected health Data (RECORD) statement. *PLoS Med* 2015;12(10):e1001885.

9. Blumenthal D, Hsiao W. Lessons from the East--China's rapidly evolving health care system. *N Engl J Med* 2015;372(14):1281-5.

10. Babiarz KS, Miller G, Yi H, et al. New evidence on the impact of China's New

For peer review only

For peer review only - http://bmjopen.bmj.com/site/about/guidelines.xhtml

Rural Cooperative Medical Scheme and its implications for rural primary healthcare: multivariate difference-in-difference analysis. *BMJ* 2010;341:c5617.

11. Hicks KA, Tcheng JE, Bozkurt B, et al. 2014 ACC/AHA Key Data Elements and Definitions for Cardiovascular Endpoint Events in Clinical Trials: A Report of the American College of Cardiology/American Heart Association Task Force on Clinical Data Standards (Writing Committee to Develop Cardiovascular Endpoints Data Standards). *Circulation* 2015;132(4):302-61.
12. Wang JB, Gu MJ, Shen P, et al. Body Mass Index and Mortality: A 10-Year Prospective Study in China. *Sci Rep* 2016;6:31609.
13. Solenkova NV, Newman JD, Berger JS, et al. Metal pollutants and cardiovascular disease: mechanisms and consequences of exposure. *Am Heart J* 2014;168(6):812-22.
14. Zhang Y, Ji X, Ku T, et al. Heavy metals bound to fine particulate matter from northern China induce season-dependent health risks: A study based on myocardial toxicity. *Environ Pollut* 2016;216:380-90.
15. Chen R, Wang C, Meng X, et al. Both low and high temperature may increase the risk of stroke mortality. *Neurology* 2013;81(12):1064-70.
16. Taljaard M, Tuna M, Bennett C, et al. Cardiovascular Disease Population Risk Tool (CVDPoRT): predictive algorithm for assessing CVD risk in the community setting. A study protocol. *BMJ Open* 2014;4(10):e006701.
17. Collins GS, Reitsma JB, Altman DG, et al. Transparent reporting of a multivariable prediction model for individual prognosis or diagnosis (TRIPOD): the TRIPOD statement. *BMJ* 2015;350:g7594.
18. Woodward M, Tunstall-Pedoe H, Peters SA. Graphics and statistics for cardiology: clinical prediction rules. *Heart* 2017;103(7):538-45.
19. D'Agostino RB, Sr., Vasan RS, Pencina MJ, et al. General cardiovascular risk profile for use in primary care: the Framingham Heart Study. *Circulation* 2008;117(6):743-53.

For peer review only

For peer review only - http://bmjopen.bmj.com/site/about/guidelines.xhtml

20. Wu N, Tang X, Wu Y, et al. Cohort profile: the Fangshan Cohort Study of cardiovascular epidemiology in Beijing, China. *J Epidemiol* 2014;24(1):84-93.

21. Zhao D, Liu J, Xie W, et al. Cardiovascular risk assessment: a global perspective. *Nat Rev Cardiol* 2015;12(5):301-11.

22. Yang X, Li J, Hu D, et al. Predicting the 10-Year Risks of Atherosclerotic Cardiovascular Disease in Chinese Population: The China-PAR Project (Prediction for ASCVD Risk in China). *Circulation* 2016;134(19):1430-40.

23. Chung SC, Sundstrom J, Gale CP, et al. Comparison of hospital variation in acute myocardial infarction care and outcome between Sweden and United Kingdom: population based cohort study using nationwide clinical registries. *BMJ* 2015;351:h3913.

24. Goldstein BA, Navar AM, Pencina MJ, et al. Opportunities and challenges in developing risk prediction models with electronic health records data: a systematic review. *J Am Med Inform Assoc* 2017;24(1):198-208.

25. Wang Y, Cui L, Ji X, et al. The China National Stroke Registry for patients with acute cerebrovascular events: design, rationale, and baseline patient characteristics. *Int J Stroke* 2011;6(4):355-61.

26. Milea D, Azmi S, Reginald P, et al. A review of accessibility of administrative healthcare databases in the Asia-Pacific region. *J Mark Access Health Policy* 2015;3.

27. Birkhead GS, Klompas M, Shah NR. Uses of electronic health records for public health surveillance to advance public health. *Annu Rev Public Health* 2015;36:345-59.

28. Yusuf S, Hawken S, Ounpuu S, et al. Effect of potentially modifiable risk factors associated with myocardial infarction in 52 countries (the INTERHEART study): case-control study. *Lancet* 2004;364(9438):937-52.

29. O'Donnell MJ, Chin SL, Rangarajan S, et al. Global and regional effects of potentially modifiable risk factors associated with acute stroke in 32

For peer review only

For peer review only - http://bmjopen.bmj.com/site/about/guidelines.xhtml

countries (INTERSTROKE): a case-control study. *Lancet* 2016;388(10046):761-75.

30. Yang Q, Zhong Y, Gillespie C, et al. Assessing potential population impact of statin treatment for primary prevention of atherosclerotic cardiovascular diseases in the USA: population-based modelling study. *BMJ Open* 2017;7(1):e011684.
31. Jutte DP, Roos LL, Brownell MD. Administrative record linkage as a tool for public health research. *Annu Rev Public Health* 2011;32:91-108.
32. Herrett E, Shah AD, Boggon R, et al. Completeness and diagnostic validity of recording acute myocardial infarction events in primary care, hospital care, disease registry, and national mortality records: cohort study. *BMJ* 2013;346:f2350.
33. Kush R, Goldman M. Fostering responsible data sharing through standards. *N Engl J Med* 2014;370(23):2163-5.
34. Zhang Q, Zhao D, Xie W, et al. Recent Trends in Hospitalization for Acute Myocardial Infarction in Beijing: Increasing Overall Burden and a Transition From ST-Segment Elevation to Non-ST-Segment Elevation Myocardial Infarction in a Population-Based Study. *Medicine (Baltimore)* 2016;95(5):e2677.

For peer review only

Acknowledgments

The authors thank the Health and Family Planning Bureau of Yinzhou District for providing access to the administrative databases used in the study. We thank Analisa Avila, ELS, from Liwen Bianji, Edanz Group China (www.liwenbianji.cn/ac), for editing the English text of a draft of this manuscript.

Authors' contributions

HL, XT, and PG drafted the manuscript. HL, XT, PS, and PG conceived and designed the study. DZ, JW, JZ, PL, and YS made substantial contributions to the study design. HL, JW, and PS are responsible for study coordination; XT, PS, and JZ are responsible for data quality control; DZ, PL, and YS are responsible for data wrangling; XT, DZ, YS, and PG are responsible for data analysis. All authors contributed to the writing of the study protocol in an iterative manner, and have read and approved the final manuscript.

Funding

This study is supported by the National Natural Science Foundation of China (91546120, 81573226), Beijing Natural Science Foundation (7162107), and the National Thousand Talents Program for Distinguished Young Scholars, China (QNQR201501).

Competing interests

None declared.

For peer review only

Tables

Table 1 List of core risk factors for cardiovascular disease in the CHERRY study

	Population census and registered health insurance database	Health checks database	Disease surveillance and management database	Outpatient/inpatient EMR database (including laboratory testing)	Charge and claims database	Environmental monitoring database
**Individual-level measurements**						
• Date of entry of first registration	+					
• Date of the measurements		*	*	*	*	
• Date of birth, sex and ethnic groups	+	*		*	*	
• Marital status, education, occupation and socio-economic status (household income, living space, etc.)	+			*		
• Smoking and alcohol use (current/former/never; amount/duration etc.)	+	*	*			
• Physical activity	+	*	*			
• Weight, height, waist and hip circumference	+	*	*			
• History of hypertension and diabetes mellitus	+	*	*	*		
• Prior history of coronary heart disease (in particular myocardial infarction and angina), stroke, transient ischemic attack (TIA), and peripheral vascular disease (PVD)	+	*	*	*		
• Family history of diseases	+		+			
• Systolic and diastolic blood pressure	+	*	*			
• Metabolic factors (including fasting glucose, post-load glucose, and glycosylated hemoglobin)		*	*	*		
• Lipid profiles: total, high- and low-density lipoprotein cholesterol; triglycerides (including information about fasting status at time blood samples were taken);		*	*	*		

For peer review only

For peer review only - http://bmjopen.bmj.com/site/about/guidelines.xhtml

• Blood urea nitrogen, creatinine, uric acid	*	*	*		
• Use of cardiovascular medications (including anti-hypertensive drugs, 'statins', fibrates) and other medications (e.g., hypoglycemic agents, hormone replacement therapy)		*	*	*	
• Inflammatory markers (including homocysteine, C-reactive protein, fibrinogen, albumin, interleukin-6 and the leucocyte count)	*		*		
• Haemostatic factors (including von-Willebrand factor and fibrin D-dimer)	*		*		
• Novel CVD related markers (e.g. NT-proBNP)			*		
• Electrocardiogram (ECG)	*		*		
• Cardiovascular imaging information (e.g. progression of coronary artery calcium, carotid intima-media thickness)	*		*		
• Urine Albumin-to-Creatinine Ratio (UACR)	*	*	*		
• Cost of outpatient or inpatient admission (including fees for diagnosis, prescription, laboratory test, surgery etc.)			*	*	
**Environmental and ecological data**					
• Date and region of surveillance					*
• Air temperature and precipitation					*
• Particles with aerodynamic diameter <2.5 µm ($PM_{2.5}$)					*
• Heavy metal concentration in water (lead, cadmium, mercury and arsenic)					*

(+) indicates the database has only one record for the measurements.
(*) indicates the database has multiple records for the measurements.
Note: Health checks database refers to Health checks for New Rural Cooperative Medical Scheme, Health checks for elderly people, and Health checks for adults with hypertension and diabetes in **Figure 2**.

For peer review only

For peer review only - http://bmjopen.bmj.com/site/about/guidelines.xhtml

Table 2 Definitions of major outcomes in the CHERRY study

Events of interest	ICD-10 Code
**Primary events of interest**	
Death due to	
Ischemic heart disease	I20-I25
Cerebrovascular disease	I60-69
Major cardiovascular disease	I00-I78
All-cause mortality	
Hospitalization with main diagnosis of	
Myocardial infarction	I21, I22
Stroke	I60, I61, I63 (excluding I63.6), I64, H34.1
Congestive heart failure	I50
Outpatient visit with main diagnosis of	
Hypertension*	I10, I11, I12, I13, I15
Diabetes mellitus†	E10, E11, E13, E14
Cardiovascular disease	I00-I99
**Secondary events of interest (fatal and non-fatal)**	
ST-Elevation Myocardial Infarction (STEMI)	I21.0, I21.1, I21.2, I21.3, I22.0, I22.1, I22.8
Non-ST segment elevation myocardial infarction (NSTEMI)	I21.4
Ischemic stroke	I63, I64, H34.1 (excluding I63.6)
Hemorrhagic stroke	I60, I61
Transient ischemic attack	G45 (excluding G45.4), H34.0
Atrial fibrillation	I48
Aortic aneurysm / aortic dissection	I71
Peripheral artery disease	I70.2, I73.9, I74.3, I74.4

* Hypertension includes either registered in the hypertension management system, or self-reported history of hypertension during health checks, or hospital admission with a diagnosis of hypertension.

† Diabetes mellitus includes either registered in the diabetes management system, or self-reported history of diabetes during health checks, or hospital admission with a diagnosis of diabetes.

Note: Majority of ICD-10 codes were selected according to the published study: "The Cardiovascular Health in Ambulatory Care Research Team (CANHEART): using big data to measure and improve cardiovascular health and healthcare services." by Tu JV, *et al.*, 2015, *Circulation: Cardiovascular Quality and Outcomes*, 8, p. 208.

For peer review only - http://bmjopen.bmj.com/site/about/guidelines.xhtml

Copyright 2015 by the American Heart Association, Inc. Adapted with permission (License Number: 4252490297412). However, codes for the STEMI and NSTEMI were modified based on the study in China.[34]

For peer review only

For peer review only - http://bmjopen.bmj.com/site/about/guidelines.xhtml

Table 3 Characteristics of participants in the CHERRY study, by sex

	Men		Women		Overall	
	***N*=512,043**	**% having at least one measurement**	***N*=541,522**	**% having at least one measurement**	***N*=1,053,565**	**% having at least one measurement**
**Age of first registration** (years)	40.17 ± 15.17	100%	39.24 ± 15.56	100%	39.69 ± 15.38	100%
**Region** [n (%)]		98.93%		98.45%		98.68%
Rural	354,524(69.99%)		364,120(68.30%)		718,644(69.12%)	
Urban	152,038(30.01%)		169,017(31.70%)		321,055(30.88%)	
**Education** [n (%)]		79.05%		79.10%		79.07%
Junior high school or lower	324,438(80.16%)		352,534(82.30%)		676,972(81.26%)	
Senior high school or higher	80,307 (19.84%)		75,801(17.70%)		156,108(18.74%)	
**Smoking status** [n (%)]		88.79%		85.60%		87.15%
Never smoker	271,870(59.80%)		440,834(95.11%)		712,704(77.62%)	
Former smoker	23,099(5.08%)		2,828(0.61%)		25,927(2.82%)	
Current smoker	159,664(35.12%)		19,861(4.28%)		179,525(19.55%)	
**Alcohol use** [n (%)]		88.74%		85.57%		87.11%
≥3 days per week	87,064(19.16%)		3,608(0.78%)		90,672(9.88%)	
<3 days per week	43,811(9.64%)		3,713(0.80%)		47,524(5.18%)	
Never drinker	323,518(71.20%)		456,059(98.42%)		779,577(84.94%)	
**Body mass index (BMI)** [kg/m^2]	22.71 ± 2.32	86.97%	22.25 ± 2.57	84.06%	22.47 ± 2.46	85.47%
**Systolic blood pressure** [mmHg]	128.09 ± 14.56	41.37%	126.16 ± 15.11	40.53%	127.11 ± 14.87	40.94%
**Diastolic blood pressure** [mmHg]	79.89 ± 9.76	41.35%	78.13 ± 10.02	40.52%	79.00 ± 9.93	40.93%

For peer review only - http://bmjopen.bmj.com/site/about/guidelines.xhtml

Figure legends

Figure 1 Study location for the CHinese Electronic Health Records Research in Yinzhou (CHERRY) study

Figure 2 Data sources for establishing the CHinese Electronic health Records Research in Yinzhou (CHERRY) cohort

Notes: Although the main focus of the CHERRY study is on adults, data sources of infants, children, and pregnant women are included in the health information system, e.g. birth weight from birth certificates. However, birth records are generally not available for adults who were already over 18 years old in 2009. Maternal exposures from antenatal examination records, i.e. gestational hypertension or diabetes, were recorded in the system which can be potential risk factors for CVD prediction in women. However, a further ethics review process is required to extract maternal information in CHERRY.

For peer review only - http://bmjopen.bmj.com/site/about/guidelines.xhtml

Supplementary files

Figure S1. Administrative databases for the CHERRY cohort

Note: Datasets listed in green in the system are currently not included in the CHERRY cohort.

Table S1. Description of CHERRY study data sources and dataset codes

Table S2. Information on cardiovascular disease diagnosis in different data sources for the CHERRY study

For peer review only

For peer review only - http://bmjopen.bmj.com/site/about/guidelines.xhtml

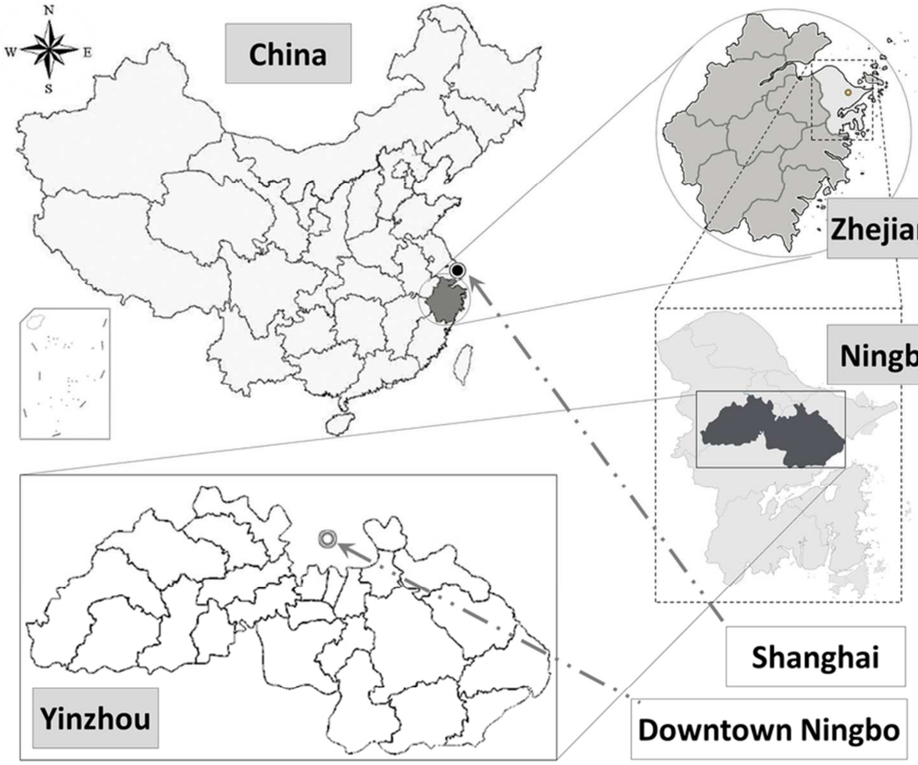

Study location for the CHinese Electronic Health Records Research in Yinzhou (CHERRY)

80x64mm (300 x 300 DPI)

For peer review only

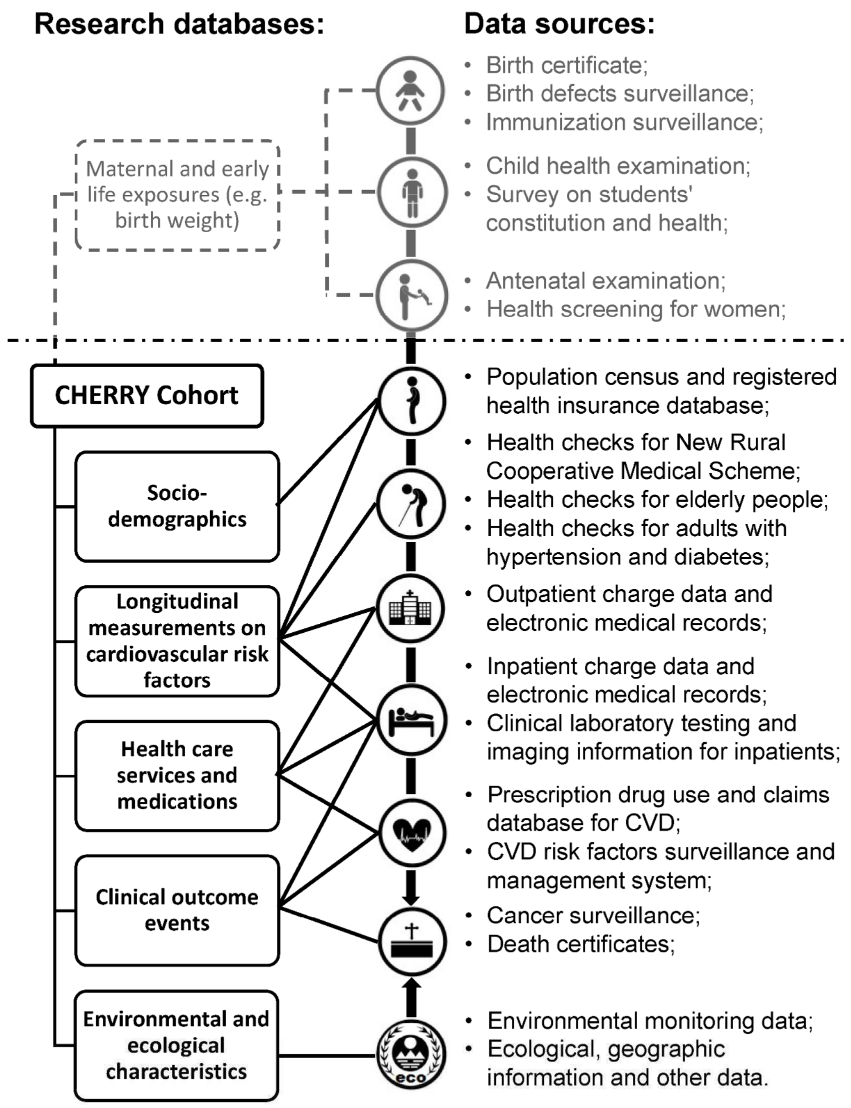

Notes: Although the main focus of the CHERRY study is on adults, data sources of infants, children, and pregnant women are included in the health information system, e.g. birth weight from birth certificates. However, birth records are generally not available for adults who were already over 18 years old in 2009. Maternal exposures from antenatal examination records, i.e. gestational hypertension or diabetes, were recorded in the system which can be potential risk factors for CVD prediction in women. However, a further ethics review process is required to extract maternal information in CHERRY.

210x298mm (300 x 300 DPI)

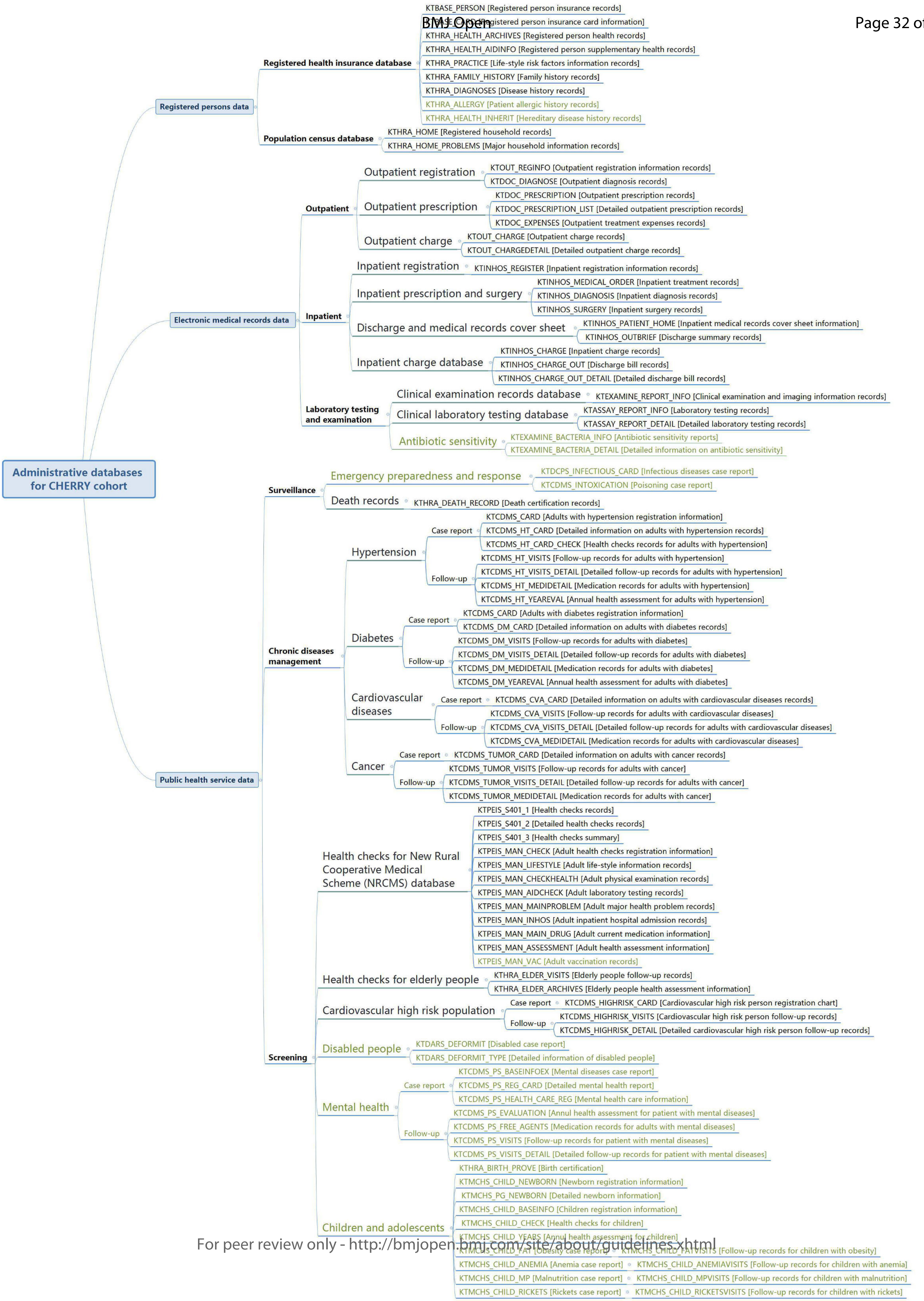
Administrative databases for CHERRY cohort
Registered persons data
Registered health insurance database
KTBASE_PERSON [Registered person insurance records]
gistered person insurance card information]
KTHRA_HEALTH_ARCHIVES [Registered person health records]
KTHRA_HEALTH_AIDINFO [Registered person supplementary health records]
KTHRA_PRACTICE [Life-style risk factors information records]
KTHRA_FAMILY_HISTORY [Family history records]
KTHRA_DIAGNOSES [Disease history records]
KTHRA_ALLERGY [Patient allergic history records]
KTHRA_HEALTH_INHERIT [Hereditary disease history records]
Population census database
KTHRA_HOME [Registered household records]
KTHRA_HOME_PROBLEMS [Major household information records]
Electronic medical records data
Outpatient
Outpatient registration
KTOUT_REGINFO [Outpatient registration information records]
KTDOC_DIAGNOSE [Outpatient diagnosis records]
Outpatient prescription
KTDOC_PRESCRIPTION [Outpatient prescription records]
KTDOC_PRESCRIPTION_LIST [Detailed outpatient prescription records]
KTDOC_EXPENSES [Outpatient treatment expenses records]
Outpatient charge
KTOUT_CHARGE [Outpatient charge records]
KTOUT_CHARGEDETAIL [Detailed outpatient charge records]
Inpatient
Inpatient registration
KTINHOS_REGISTER [Inpatient registration information records]
Inpatient prescription and surgery
KTINHOS_MEDICAL_ORDER [Inpatient treatment records]
KTINHOS_DIAGNOSIS [Inpatient diagnosis records]
KTINHOS_SURGERY [Inpatient surgery records]
Discharge and medical records cover sheet
KTINHOS_PATIENT_HOME [Inpatient medical records cover sheet information]
KTINHOS_OUTBRIEF [Discharge summary records]
Inpatient charge database
KTINHOS_CHARGE [Inpatient charge records]
KTINHOS_CHARGE_OUT [Discharge bill records]
KTINHOS_CHARGE_OUT_DETAIL [Detailed discharge bill records]
Laboratory testing and examination
Clinical examination records database
KTEXAMINE_REPORT_INFO [Clinical examination and imaging information records]
Clinical laboratory testing database
KTASSAY_REPORT_INFO [Laboratory testing records]
KTASSAY_REPORT_DETAIL [Detailed laboratory testing records]
Antibiotic sensitivity
KTEXAMINE_BACTERIA_INFO [Antibiotic sensitivity reports]
KTEXAMINE_BACTERIA_DETAIL [Detailed information on antibiotic sensitivity]
Public health service data
Surveillance
Emergency preparedness and response
KTDCPS_INFECTIOUS_CARD [Infectious diseases case report]
KTCDMS_INTOXICATION [Poisoning case report]
Death records
KTHRA_DEATH_RECORD [Death certification records]
Chronic diseases management
Hypertension
Case report
KTCDMS_CARD [Adults with hypertension registration information]
KTCDMS_HT_CARD [Detailed information on adults with hypertension records]
KTCDMS_HT_CARD_CHECK [Health checks records for adults with hypertension]
Follow-up
KTCDMS_HT_VISITS [Follow-up records for adults with hypertension]
KTCDMS_HT_VISITS_DETAIL [Detailed follow-up records for adults with hypertension]
KTCDMS_HT_MEDIDETAIL [Medication records for adults with hypertension]
KTCDMS_HT_YEAREVAL [Annual health assessment for adults with hypertension]
Diabetes
Case report
KTCDMS_CARD [Adults with diabetes registration information]
KTCDMS_DM_CARD [Detailed information on adults with diabetes records]
Follow-up
KTCDMS_DM_VISITS [Follow-up records for adults with diabetes]
KTCDMS_DM_VISITS_DETAIL [Detailed follow-up records for adults with diabetes]
KTCDMS_DM_MEDIDETAIL [Medication records for adults with diabetes]
KTCDMS_DM_YEAREVAL [Annual health assessment for adults with diabetes]
Cardiovascular diseases
Case report
KTCDMS_CVA_CARD [Detailed information on adults with cardiovascular diseases records]
Follow-up
KTCDMS_CVA_VISITS [Follow-up records for adults with cardiovascular diseases]
KTCDMS_CVA_VISITS_DETAIL [Detailed follow-up records for adults with cardiovascular diseases]
KTCDMS_CVA_MEDIDETAIL [Medication records for adults with cardiovascular diseases]
Cancer
Case report
KTCDMS_TUMOR_CARD [Detailed information on adults with cancer records]
Follow-up
KTCDMS_TUMOR_VISITS [Follow-up records for adults with cancer]
KTCDMS_TUMOR_VISITS_DETAIL [Detailed follow-up records for adults with cancer]
KTCDMS_TUMOR_MEDIDETAIL [Medication records for adults with cancer]
Screening
Health checks for New Rural Cooperative Medical Scheme (NRCMS) database
KTPEIS_S401_1 [Health checks records]
KTPEIS_S401_2 [Detailed health checks records]
KTPEIS_S401_3 [Health checks summary]
KTPEIS_MAN_CHECK [Adult health checks registration information]
KTPEIS_MAN_LIFESTYLE [Adult life-style information records]
KTPEIS_MAN_CHECKHEALTH [Adult physical examination records]
KTPEIS_MAN_AIDCHECK [Adult laboratory testing records]
KTPEIS_MAN_MAINPROBLEM [Adult major health problem records]
KTPEIS_MAN_INHOS [Adult inpatient hospital admission records]
KTPEIS_MAN_MAIN_DRUG [Adult current medication information]
KTPEIS_MAN_ASSESSMENT [Adult health assessment information]
KTPEIS_MAN_VAC [Adult vaccination records]
Health checks for elderly people
KTHRA_ELDER_VISITS [Elderly people follow-up records]
KTHRA_ELDER_ARCHIVES [Elderly people health assessment information]
Cardiovascular high risk population
Case report
KTCDMS_HIGHRISK_CARD [Cardiovascular high risk person registration chart]
Follow-up
KTCDMS_HIGHRISK_VISITS [Cardiovascular high risk person follow-up records]
KTCDMS_HIGHRISK_DETAIL [Detailed cardiovascular high risk person follow-up records]
Disabled people
KTDARS_DEFORMIT [Disabled case report]
KTDARS_DEFORMIT_TYPE [Detailed information of disabled people]
Mental health
Case report
KTCDMS_PS_BASEINFOEX [Mental diseases case report]
KTCDMS_PS_REG_CARD [Detailed mental health report]
KTCDMS_PS_HEALTH_CARE_REG [Mental health care information]
Follow-up
KTCDMS_PS_EVALUATION [Annul health assessment for patient with mental diseases]
KTCDMS_PS_FREE_AGENTS [Medication records for adults with mental diseases]
KTCDMS_PS_VISITS [Follow-up records for patient with mental diseases]
KTCDMS_PS_VISITS_DETAIL [Detailed follow-up records for patient with mental diseases]
Children and adolescents
KTHRA_BIRTH_PROVE [Birth certification]
KTMCHS_CHILD_NEWBORN [Newborn registration information]
KTMCHS_PG_NEWBORN [Detailed newborn information]
KTMCHS_CHILD_BASEINFO [Children registration information]
KTMCHS_CHILD_CHECK [Health checks for children]
KTMCHS_CHILD_YEARS [Annul health assessment for children]
KTMCHS_CHILD_FAT [Obesity case report]
KTMCHS_CHILD_FATVISITS [Follow-up records for children with obesity]
KTMCHS_CHILD_ANEMIA [Anemia case report]
KTMCHS_CHILD_ANEMIAVISITS [Follow-up records for children with anemia]
KTMCHS_CHILD_MP [Malnutrition case report]
KTMCHS_CHILD_MPVISITS [Follow-up records for children with malnutrition]
KTMCHS_CHILD_RICKETS [Rickets case report]
KTMCHS_CHILD_RICKETSVISITS [Follow-up records for children with rickets]

Supplemental files

Table S1. Description of CHERRY study data sources and dataset codes

Research databases	Data sources	Dataset codes	Description
Socio-demographics	Registered health insurance database	KTBASE_PERSON	Registered person insurance records
		KTBASE_CARD	Registered person insurance card information
		KTHRA_HEALTH_ARCHIVES	Registered person health records
		KTHRA_HEALTH_AIDINFO	Registered person supplementary health records
		KTHRA_PRACTICE	Life-style risk factors information records
		KTHRA_FAMILY_HISTORY	Family history records
		KTHRA_DIAGNOSES	Disease history records
	Population census database	KTHRA_HOME	Registered household records
		KTHRA_HOME_PROBLEMS	Major household information records

For peer review only - http://bmjopen.bmj.com/site/about/guidelines.xhtml

Longitudinal measurements on cardiovascular risk factors	Health checks for New Rural Cooperative Medical Scheme (NRCMS) database	KTPEIS_S401_1	Health checks records
		KTPEIS_S401_2	Detailed health checks records
		KTPEIS_S401_3	Health checks summary
		KTPEIS_MAN_CHECK	Adult health checks registration information
		KTPEIS_MAN_LIFESTYLE	Adult life-style information records
		KTPEIS_MAN_CHECKHEALTH	Adult physical examination records
		KTPEIS_MAN_AIDCHECK	Adult laboratory testing records
		KTPEIS_MAN_MAINPROBLEM	Adult major health problem records
		KTPEIS_MAN_INHOS	Adult inpatient hospital admission records
		KTPEIS_MAN_MAIN_DRUG	Adult current medication information
		KTPEIS_MAN_ASSESSMENT	Adult health assessment information

For peer review only - http://bmjopen.bmj.com/site/about/guidelines.xhtml

For peer review only

	Health checks for elderly people database	KTHRA_ELDER_VISITS	Elderly people follow-up records
		KTHRA_ELDER_ARCHIVES	Elderly people health assessment information
	Health checks for adults with hypertension database	KTCDMS_CARD	Adults with hypertension registration information
		KTCDMS_HT_CARD	Detailed information on adults with hypertension records
		KTCDMS_HT_CARD_CHECK	Health checks records for adults with hypertension
		KTCDMS_HT_VISITS	Follow-up records for adults with hypertension
		KTCDMS_HT_VISITS_DETAIL	Detailed follow-up records for adults with hypertension
		KTCDMS_HT_MEDIDETAIL	Medication records for adults with hypertension
		KTCDMS_HT_YEAREVAL	Annual health assessment for adults with hypertension
	Health checks for adults with diabetes database	KTCDMS_CARD	Adults with diabetes registration information
		KTCDMS_DM_CARD	Detailed information on adults with diabetes records

For peer review only - http://bmjopen.bmj.com/site/about/guidelines.xhtml

	KTCDMS_DM_VISITS	Follow-up records for adults with diabetes
	KTCDMS_DM_VISITS_DETAIL	Detailed follow-up records for adults with diabetes
	KTCDMS_DM_MEDIDETAIL	Medication records for adults with diabetes
	KTCDMS_DM_YEAREVAL	Annual health assessment for adults with diabetes
Cardiovascular high-risk population	KTCDMS_HIGHRISK_CARD	Cardiovascular high-risk person registration chart
	KTCDMS_HIGHRISK_VISITS	Cardiovascular high-risk person follow-up records
	KTCDMS_HIGHRISK_DETAIL	Detailed cardiovascular high-risk person follow-up records
Clinical examination records database	KTEXAMINE_REPORT_INFO	Clinical examination and imaging information records
Clinical laboratory testing database	KTASSAY_REPORT_INFO	Laboratory testing records
	KTASSAY_REPORT_DETAIL	Detailed laboratory testing records

For peer review only - http://bmjopen.bmj.com/site/about/guidelines.xhtml
For peer review only

Health care services and medications	Outpatient medical records database	KTOUT_REGINFO	Outpatient registration information records
		KTDOC_DIAGNOSE	Outpatient diagnosis records
	Outpatient prescription database	KTDOC_PRESCRIPTION	Outpatient prescription records
		KTDOC_PRESCRIPTION_LIST	Detailed outpatient prescription records
		KTDOC_EXPENSES	Outpatient treatment expenses records
	Outpatient charge database	KTOUT_CHARGE	Outpatient charge records
		KTOUT_CHARGEDETAIL	Detailed outpatient charge records
	Inpatient medical records database	KTINHOS_REGISTER	Inpatient registration information records
		KTINHOS_PATIENT_HOME	Inpatient medical records cover sheet information
		KTINHOS_OUTBRIEF	Discharge summary records

For peer review only

For peer review only - http://bmjopen.bmj.com/site/about/guidelines.xhtml

	Inpatient prescription database	KTINHOS_MEDICAL_ORDER	Inpatient treatment records
		KTINHOS_DIAGNOSIS	Inpatient diagnosis records
		KTINHOS_SURGERY	Inpatient surgery records
	Inpatient charge database	KTINHOS_CHARGE	Inpatient charge records
		KTINHOS_CHARGE_OUT	Discharge bill records
		KTINHOS_CHARGE_OUT_DETAIL	Detailed discharge bill records
Clinical outcome events	Cardiovascular diseases surveillance and management system	KTCDMS_CVA_CARD	Detailed information on adults with cardiovascular disease records
		KTCDMS_CVA_VISITS	Follow-up records for adults with cardiovascular diseases
		KTCDMS_CVA_VISITS_DETAIL	Detailed follow-up records for adults with cardiovascular diseases
		KTCDMS_CVA_MEDIDETAIL	Medication records for adults with cardiovascular diseases

For peer review only

Cancer surveillance database	KTCDMS_TUMOR_CARD	Detailed information on adults with cancer records
	KTCDMS_TUMOR_VISITS	Follow-up records for adults with cancer
	KTCDMS_TUMOR_VISITS_DETAIL	Detailed follow-up records for adults with cancer
	KTCDMS_TUMOR_MEDIDETAIL	Medication records for adults with cancer
Database of death records	KTHRA_DEATH_RECORD	Death certificates

For peer review only

For peer review only - http://bmjopen.bmj.com/site/about/guidelines.xhtml

Table S2. Information on cardiovascular disease diagnosis in different data sources for the CHERRY study

	Clinical diagnosis	ICD-10 Code	Clinical symptom		Neurological examination	Cerebrospinal fluid (CSF) analysis	Serum enzyme tests	Electrocardiogram (ECG)	Electroencephalogram (EEG)	Coronary computed tomography angiography (CTA)	Computed tomography (CT) / magnetic resonance imaging (MRI)		Autopsy
			Brief	Detail							Summary	Imaging	
Disease surveillance / Death certificates (gold standard)	√	√	√				√	√	√	√	√		√
Disease management database (primary care)	√	√	√	√			√	√	√		√		
Electronic medical records database (hospital care)	√	√	√	√	√	√	√	√	√	√	√	√	
Health insurance database	√	√											

For peer review only
